# Quantitative Prediction of Rate Constants for Aqueous Racemization To Avoid Pointless Stereoselective Syntheses

**DOI:** 10.1002/anie.201709163

**Published:** 2017-11-15

**Authors:** Andrew Ballard, Hiwa O. Ahmad, Stefania Narduolo, Lucy Rosa, Nikki Chand, David A. Cosgrove, Peter Varkonyi, Nabil Asaad, Simone Tomasi, Niklaas J. Buurma, Andrew G. Leach

**Affiliations:** ^1^ Physical Organic Chemistry Centre School of Chemistry Cardiff University Main Building, Park Place, Cardiff CF10 3AT UK; ^2^ Pharmaceutical Chemistry Department College of Pharmacy Hawler Medical University Erbil Kurdistan Region Iraq; ^3^ AstraZeneca Pharmaceuticals, Mereside Alderley Park Macclesfield SK10 4TG UK; ^4^ AstraZeneca R+D Pepparedsleden 1 43183 Mölndal Sweden; ^5^ AstraZeneca Charter Way, Silk Road Business Park Macclesfield SK10 2NA UK; ^6^ School of Pharmacy and Biomolecular Sciences Liverpool John Moores University Liverpool L3 3AF UK

**Keywords:** computational chemistry, drug design, kinetics, racemization, stereoselective synthesis

## Abstract

Racemization has a large impact upon the biological properties of molecules but the chemical scope of compounds with known rate constants for racemization in aqueous conditions was hitherto limited. To address this remarkable blind spot, we have measured the kinetics for racemization of 28 compounds using circular dichroism and ^1^H NMR spectroscopy. We show that rate constants for racemization (measured by ourselves and others) correlate well with deprotonation energies from quantum mechanical (QM) and group contribution calculations. Such calculations thus provide predictions of the second‐order rate constants for general‐base‐catalyzed racemization that are usefully accurate. When applied to recent publications describing the stereoselective synthesis of compounds of purported biological value, the calculations reveal that racemization would be sufficiently fast to render these expensive syntheses pointless.

Thalidomide racemizes in a matter of hours and yet it remains a poster child for enantioselective synthesis which would not have saved its victims.^1^ The status quo in enantioselective synthesis thus ignores the cruel blind spot that we address in this paper: racemization.

Although necessary in dynamic kinetic resolution protocols,^2^, ^3^ racemization and epimerization can cause safe compounds to become toxic or lose efficacy,^4^, ^5^, ^6^, ^7^, ^8^, ^9^, ^10^, ^11^ lead to misidentification of chiral compounds extracted from natural sources,^12^ etc. Ignoring racemization thus leads to wasted material and human resources.

Racemization is a particular problem because its detection requires chiral analytical methods.^13^, ^14^ Hence, few reports disclose rate constants for racemization under aqueous conditions.^1^, ^15^, ^16^, ^17^, ^18^, ^19^, ^20^ Chiral centers with certain combinations of substituents have been posited to be prone to general‐base‐catalyzed racemization although with little supporting data.^21^, ^22^, ^23^

We therefore classified stereogenic carbon atoms according to their attached substituents. Each substituent is identified as one of sixty types,^24^ which encompass more than 99.95 % of all such substituents in the GOSTAR database.^25^ The ten most frequently occurring substituents are listed in Figure 1; the H required for general‐base‐catalyzed racemization is prominent.^24^ Groups labelled * were selected for experimental study.

**Figure 1:**
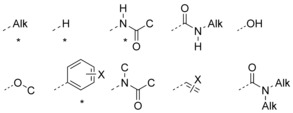
The ten substituents appearing most frequently adjacent to carbon stereogenic centers in the GOSTAR database. Alk=alkyl, C=carbon‐linked alkyl or aromatic group and X=any group. *=selected for experimental study.

Based on prevalence, earlier work,^21^, ^22^, ^23^ and chemical intuition, several compounds were selected for detailed kinetic studies.^26^, ^27^, ^28^ The rate constants for general‐base‐catalyzed racemization were derived for a range of 11 arylglycine derivatives (**1**, **2** and **3**), 12 hydantoins (**4**, **5** and **6**) and 5 thiohydantoins (**7** and **8**). Briefly, at several buffer concentrations, circular dichroism spectroscopy (CD) followed the decrease in ellipticity and/or ^1^H NMR spectroscopy the incorporation of D from deuterated buffers. The pseudo‐first‐order rate constants for these processes were corrected for hydrolysis side reactions if required.^24^ Plotting the first‐order rate constants for racemization or H/D exchange against the concentration of the basic component of the buffer yielded the second‐order rate constants for general‐base‐catalyzed racemization. These were corrected for reaction temperature and substrate protonation state.^24^

For predictive modeling, a mechanistic understanding is beneficial. Racemization of the stereogenic centers studied here could occur by either an S_E_1 or an S_E_2 mechanism. For hydantoins (e.g. **4**–**6**) both the S_E_1 and S_E_2 mechanisms have been proposed previously,^18^, ^29^ but we have shown that these reactions occur via the S_E_1 mechanism.^30^ Further, Hammett plots for **1a**‐**h** show a positive slope and better correlation with σ^−^ than σ suggesting that a negative charge is formed on the stereogenic center in the rate‐determining step of the racemization reaction, in line with an S_E_1 mechanism.^24^

The experimental data were correlated with deprotonation energies (ΔΔ*G*(R_1_,R_2_,R_3_), Scheme 1) from B3LYP/6–31+G** calculations incorporating aqueous solvation using the PCM protocol.^24^ Second‐order rate constants for general‐base‐catalyzed racemization, *k*_gb_, correlate well with ΔΔ*G*(R_1_,R_2_,R_3_) for **1**–**3** and **4**–**8**.^24^

**Scheme 1:**
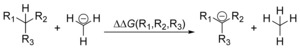



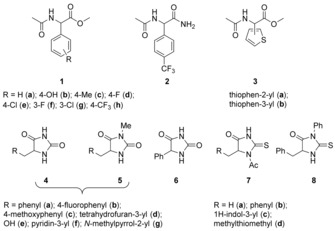


The set of compounds was supplemented with literature data for **9**‐**16**,^24^ leading to the relationship with ΔΔ*G*(R_1_,R_2_,R_3_) shown at the top of Figure 2. The line of best fit has equation log(*k*_gb_)=−0.20×ΔΔ*G*(R_1_,R_2_,R_3_) −14.28, with an *R* ^2^ value of 0.68 and root mean square error of 0.61, that is, reproducing rate constants to within a factor of approximately 4. Clopidogrel is excluded from this analysis due to large experimental uncertainties.^24^

**Figure 2:**
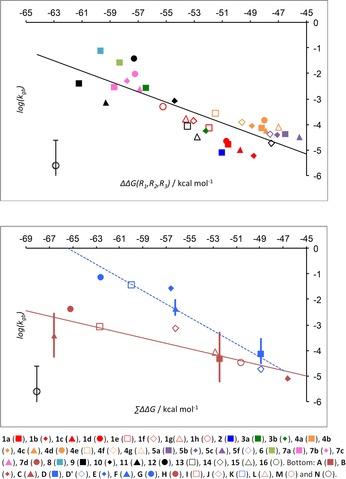
Second‐order rate constants for racemization under aqueous general‐base‐catalyzed conditions plotted against: computed ΔΔ*G*(R_1_,R_2_,R_3_) values (top) and ΣΔΔ*G* values (bottom). Clopidogrel (**16**) is excluded from the line.


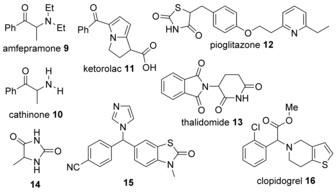


The computational procedure was extended to include a group‐contribution approach that is amenable to rapid analysis of chiral compounds and is described with examples in section S5. A simple representative (R) of each substituent type was selected and ΔΔ*G*(R_1_,R_2_,R_3_) computed with R_1_=R and R_2_=R_3_=H. These ΔΔ*G*(R,H,H) values indicate how much R stabilizes an adjacent anion. The sum ΔΔ*G*(R_1_,H,H)+ΔΔ*G*(R_2_,H,H)+ΔΔ*G*(R_3_,H,H), for the three non‐H substituents around a chiral carbon atom is referred to here as ΣΔΔ*G*. When two or three of the groups provide stabilization through charge delocalization, a cross‐conjugation correction is applied to reflect the reduced ability of the second group to stabilize the anion caused by the presence of the first.^24^

The 35 compounds studied fall into fourteen chiral carbon atom types (**A**–**N**, Table 1) that have one hydrogen attached.^1^, ^15^, ^16^, ^17^, ^18^, ^19^, ^31^ The second‐order rate constants for general‐base‐catalyzed racemization are plotted against ΣΔΔ*G* at the bottom of Figure 2. When a chiral center type is represented by more than one compound, the mean value of log(*k*_gb_) for all representatives was used and the full range of values is shown as a vertical line. This prevents any center type from dominating the linear fit.

**Table 1:** Stereogenic center types.

Center type	Non‐H substituents	Representative compounds [number]
**A**	phenyl, reversed secondary amide, ester	**1 a**–**h** [8]
**B**	phenyl, reversed secondary amide, primary amide	**2** [1]
**C**	5‐membered aromatic, reversed secondary amide, ester	**3** [2]
**D**/**D′**	alkyl, reversed secondary amide, acidic secondary amide	**D=4 a–g**, **5 a–c+f** [11] **D′=14** [1]
**E**	phenyl, reversed secondary amide, acidic secondary amide	**6** [1]
**F**	alkyl, aminothiooxo imide, acidic secondary amide	**7 a–d** [4]
**G**	alkyl, reversed secondary thioamide, acidic secondary amide	**8** [1]
**H**	ketone, dialkyl tertiary amine, alkyl	**9** [1]
**I**	ketone, primary amine, alkyl	**10** [1]
**J**	carboxylic acid, 5‐membered aromatic, alkyl	**11** [1]
**K**	thioether, alkyl, acidic secondary amide	**12** [1]
**L**	imide, alkyl, acidic secondary amide	**13** [1]
**M**	phenyl, phenyl, 5‐membered aromatic	**15** [1]
**N**	ester, dialkyl tertiary amine, phenyl	**16** [1]

For the phenylglycine esters (**A**), substituent effects can cause up to a log unit variation from the line of best fit. This is likely to be representative of general substituent effects.^24^ Grouping five‐membered aromatic rings together (**C**) masks variation of 1.6 log units, likely reflecting the more direct influence of heteroatoms in aromatic rings. A relatively diverse set of alkyl substituents in the 5‐position on a hydantoin ring (group **D**) or thiohydantoin (**F**) causes little variation in rate constants for racemization. In general, variation caused by substitution or structural variation within classes is less than two orders of magnitude and typically less than one order of magnitude.

At the bottom of Figure 2, two subgroups are apparent: those involving a cyclic anion with the potential to be aromatic and those that do not. For the non‐aromatic set (shown in red) a line of best fit with equation log(*k*_gb_)=−0.11×ΣΔΔ*G* −9.81 was found (R^2^=0.78 and RMSE=0.40 log units) and for the aromatic anion set (shown in blue) the line of best fit has equation log(*k*_gb_)=−0.26×ΣΔΔ*G* −16.95 (R^2^=0.92 and RMSE=0.39 log units). The RMSE for all compounds computed individually is 0.64 for non‐aromatic anions (excluding clopidogrel) and 0.37 for aromatic anions, that is, predictions are typically within 5‐fold. Although not a perfect guide, the group contribution approach provides an easily applied, useful and rapid filtering that can even be used for very large databases.

For the particular example of chiral pharmaceuticals, our analysis can be applied to predict half‐lives of racemization in physiological conditions. The rate constants for racemization of thalidomide at different phosphate buffer concentrations compared to that in blood suggest that, in terms of availability of catalytically active general bases, blood is approximately equivalent to a 0.15 m phosphate buffer at pH 7.2. Therefore, with *k*_gb_ predicted by the QM or group contribution method, the required half‐lives can be predicted.^1^

A comprehensive workflow has now arisen: rapid analysis with a group contribution based method can trigger quantum mechanical calculations, which in turn can trigger an experimental protocol (Figure 3). Compounds at high risk of racemization can be avoided and racemization risk can be suppressed by design.

**Figure 3:**
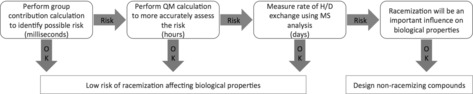
Workflow for identifying compounds at risk of racemization, in parentheses is the typical time taken to process one compound at each step.

To illustrate the degree to which racemization in aqueous conditions is an overlooked issue, we have surveyed recent editions of leading chemistry journals, using our knowledge of the group contributions, to identify several articles envisaging biological applications. This was not an exhaustive search. Compounds described were subject to group contribution calculations. It is disappointing to reveal (Table 2) that liability to racemize under physiological conditions is more commonplace than would be possible if it were properly understood and controlled, as many chemists seem to believe.

**Table 2:** Examples of potentially pointless stereoselective syntheses from recent literature.

Reference	Representative compound	ΣΔΔ*G* [kcal mol^−1^]	Predicted percentage^[a]^
32	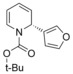	−48.8	28 %
33	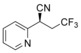	−50.7	40 %
34	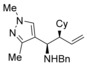	−54.1	70 %
35	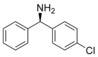	−46.9	19 %

[a] Racemized within 24 hours.

In summary, we describe an approach to quantitatively predict the racemization risk that is generally applicable and allows synthetic chemists to avoid racemization‐prone targets or understand erosion of the enantiomeric excess (*ee*). The approach allows quantitative assessment of the risk of chiral compounds of turning into racemic mixtures when used as pharmaceuticals or for other purposes.^36^

## Conflict of interest

A.G.L. is a shareholder in AstraZeneca.

## Supporting information

As a service to our authors and readers, this journal provides supporting information supplied by the authors. Such materials are peer reviewed and may be re‐organized for online delivery, but are not copy‐edited or typeset. Technical support issues arising from supporting information (other than missing files) should be addressed to the authors.

Supplementary
