# Supplementary material for: Quantitative Prediction of Rate Constants for Aqueous Racemization To Avoid Pointless Stereoselective Syntheses
Source: Angew Chem Int Ed Engl. 2017 Nov 15;57(4):982–5. doi: 10.1002/anie.201709163 (PMC5820753; doi:10.1002/anie.201709163)
Supplement: Supplementary file 1 — Supplementary [file ANIE-57-982-s001.pdf]

Angewandte Chemie
Eine Zeitschrift der Gesellschaft Deutscher Chemiker

Supporting Information

Quantitative Prediction of Rate Constants for Aqueous Racemization To Avoid Pointless Stereoselective Syntheses

Andrew Ballard, Hiwa O. Ahmad, Stefania Narduolo, Lucy Rosa, Nikki Chand, David A. Cosgrove, Peter Varkonyi, Nabil Asaad, Simone Tomasi, Niklaas J. Buurma, and Andrew G. Leach**

anie_201709163_sm_miscellaneous_information.pdf

Supporting Information

TOC

S1.	Second-order rate constants for phosphate-catalysed racemization reactions		S3
	1	N-Acetylarylglycine methyl esters	S4
	2	N-Acetyl-(4-trifluoromethylphenyl)-glycine amide	S6
	3	N-Acetylthiophenylglycine methyl esters	S7
	4	5-Substituted hydantoins	S8
	5	5-Substituted 3-*N*-methylhydantoins	S10
	6	5-Phenylhydantoin	S11
	7	5-Substituted 1-*N*-acetyl-2-thiohydantoins	S12
	8	3-*N*-phenyl-5-benzyl-2-thiohydantoin	S14
S2.	Second-order rate constants for phosphate-catalysed racemization reactions (literature data)		S15
	9	Amfepramone	S15
	10	Cathinone	S16
	11	Ketorolac	S17
	12	Pioglitazone (or Rosiglitazone)	S19
	13	Thalidomide	S20
	14	5-Methylhydantoin	S21
	15	3-methyl-6-[1-(imidazol-1-yl)-1-(4-cyanophenyl)methyl] benzothiazolinone	S22
	16	Clopidogrel	S24
S3.	Hammett plots for compounds **1a-h**		S26
S4.	Relationship between log(k_{gb}) and ΔG(R_1,R_2,R_3) for compounds **1-8**		S27
S5.	Example application of the group contribution method		S29
S6.	Program for identifying and flagging groups attached to a chiral center (and adding [Xe] at the point of attachment to the chiral center)		S30
S7.	SMARTS strings defining groups attached to a chiral center and encoded with [Xe] as the attachment point to the chiral center (generated using the program above)		S32
S8.	Quantum mechanical free energies for the representative example of each group type		S35
S9.	Quantum mechanical energies for full QM method		S37
S10.	Explanation for why k_{obs} is maximized when pH ~ pK_a		S38
S11.	Frequency of occurrence of functional group types adjacent to chiral centres in the GOSTAR database		S39
S12.	Justification of the cross-conjugation correction.		S41
S13.	References		S42

S1 second-order rate constants for phosphate-catalysed racemization reactions

In the following, the pK_a of phosphate is set to the pK_a of 6.81 as reported by Fukada for 0.01 M phosphate in 0.1 M KCl,[1] i.e. at an ionic strength of approximately 0.1 M at 25 °C unless otherwise noted. This choice ignores the effect of ionic strength on the buffer pK_a which may lead to a variation in pK_a from 7.2 to 6.6.[2] Although ionic strengths for buffers used in kinetic studies of racemization reactions have been routinely reported in the literature, it is not always clear whether the pH of buffers was adjusted before or after adjusting the ionic strength or whether the pH was assumed based on the buffer ratio. Assuming relevant pK_a values to be between 6.6 and 6.8 (i.e. most experiments carried out at an ionic strength in excess of 0.1 M) and with the majority of experiments carried out at a pH of 7.4, the error in calculated free phosphate concentrations is expected to be typically less than a factor 1.1.

For all compounds, synthesis and purification, experimental details for the kinetic studies, and analysis of the kinetic data are provided in the cited sources.

S1.1 N-Acetylarylglycine methyl esters[3]

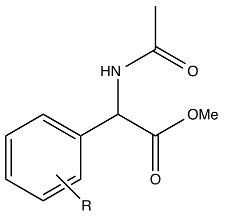

experimental data

Table S1.1.1: reaction conditions		
temperature T		37 °C
pH / pH* / pH** / pD		pH** 7.4[4]
ionic strength I / M		1.0
constant ionic strength		yes
solvent		aqueous buffers without co-solvent
process followed		H/D exchange
technique used		^{1}H NMR
relevant substrate pK_as		None relevant. Racemisation is presumed to proceed through the neutral form. Deprotonation of the amide is likely to be to a degree much less than 1%.
DOI		http://orca.cf.ac.uk/55130/1/U585519.pdf

The observed kinetics represent both H/D exchange and ester hydrolysis, as illustrated for compound **1a** in Scheme S1.1.1.

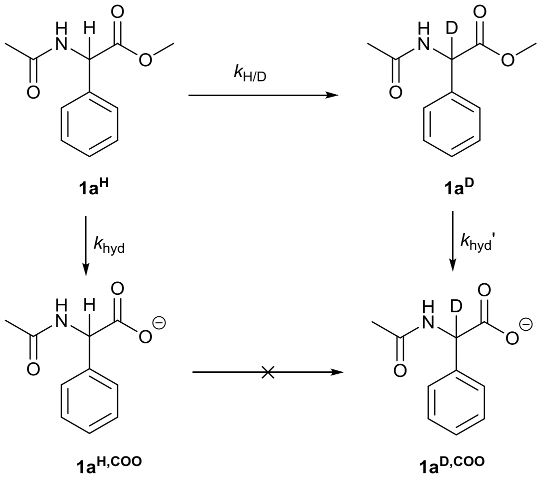

Scheme S1.1.1

^{1}H NMR spectroscopy using the aromatic ring as a reference allows us to monitor the stereogenic proton in $\mathbf{1a^H}$ and $\mathbf{1a^{H,COO}}$ while $\mathbf{1a^D} + \mathbf{1a^{D,COO}} = \mathbf{1a^H}_{t=0} - (\mathbf{1a^H}$ and $\mathbf{1a^{H,COO}})$.

The kinetic scheme (Scheme S1.1.1) for a single starting material undergoing several reactions in parallel results in a single observed rate constant k_{obs} (with $k_{obs} = k_{H/D} + k_{hyd}$) which is obtained from global analysis of all measurable relative concentrations (Figure S1.1.1).

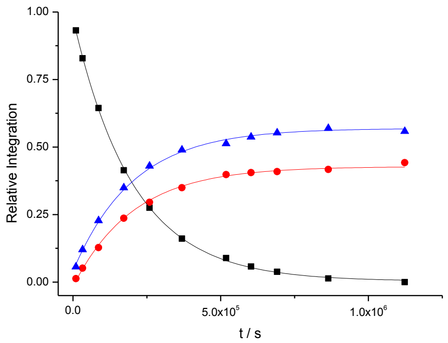

Figure S1.1.1: Relative integration of (■) [**1a**^{H}], (●) [**1a**^{H,COO}] and (▲){[**1a**^{D}] + [**1a**^{D,COO}]} as a function of time in 0.2 M D_2O phosphate buffer, I = 1 M, at 37 °C.

The individual rate constants for H/D exchange and hydrolysis reactions are determined from the relative final amounts of **1a**^{H,COO} and {**1a**^{D} + **1a**^{D,COO}}. The ratio of these products is equal to the ratio of the relative rate constants k_{deut} and k_{hyd} .

$$\frac{k_{\mathrm{H/D}}}{k_{\mathrm{hyd}}} = \frac{S_{\mathrm{f\ (deut)}}}{S_{\mathrm{f\ (hyd)}}} \therefore k_{hyd} = k_{\mathrm{H/D}} \cdot \frac{S_{\mathrm{f\ (hyd)}}}{S_{\mathrm{f\ (deut)}}}$$

Where $S_{f\,(deut)}$ is the final proportion of compound deuterated at the stereogenic centre and $S_{f\,(hyd)}$ is the final proportion of carboxylate protonated at the stereogenic centre. The rate constant $k_{H/D}$ is then obtained from the following relationship.

$$k_{\mathrm{obs}} = k_{\mathrm{H/D}} \cdot \left\{ \frac{S_{\mathrm{f\ (hyd)}}}{S_{\mathrm{f\ (deut)}}} + 1 \right\}$$

Dependence of rate constant of deuteration upon [HPO_4^{2-}] indicates that for
Rate of H/D exchange = $k_{2,\,neut,\,HPO4\_2-}$ × [HPO_4^{2-}] × [neutral substrate]:
R=H $k_{2,\,neut,\,HPO4\_2-}$ = 1.64 x 10^{-5} M^{-1}s^{-1}
R=p-OH $k_{2,\,neut,\,HPO4\_2-}$ = 6.01 x 10^{-6} M^{-1}s^{-1}
R=p-Me $k_{2,\,neut,\,HPO4\_2-}$ = 1.03 x 10^{-5} M^{-1}s^{-1}
R=p-F $k_{2,\,neut,\,HPO4\_2-}$ = 2.20 x 10^{-5} M^{-1}s^{-1}
R=p-Cl $k_{2,\,neut,\,HPO4\_2-}$ = 7.47 x 10^{-5} M^{-1}s^{-1}
R=m-F $k_{2,\,neut,\,HPO4\_2-}$ = 1.40 x 10^{-4} M^{-1}s^{-1}
R=m-Cl $k_{2,\,neut,\,HPO4\_2-}$ = 1.60 x 10^{-4} M^{-1}s^{-1}
R=p-CF_3 $k_{2,\,neut,\,HPO4\_2-}$ = 5.15 x 10^{-4} M^{-1}s^{-1}

These rate constants are taken individually for the full QM calculations and plotted as a geometric mean for the group contribution method.
computed values
R=H ΔΔG = -50.6 kcal/mol
R=p-OH ΔΔG = -48.8 kcal/mol
R=p-Me ΔΔG = -49.7 kcal/mol
R=p-F ΔΔG = -50.7 kcal/mol
R=p-Cl ΔΔG = -52.0 kcal/mol
R=m-F ΔΔG = -53.0 kcal/mol
R=m-Cl ΔΔG = -53.6 kcal/mol
R=p-CF_3 ΔΔG = -55.2 kcal/mol

Sum of group contributions = -38.6 (ester) -8.9 (reverse secondary amide) -19.9 (benzene) +15.0 (cross-conjugation correction) = -52.4 kcal/mol

S1.2 N-Acetyl-(4-trifluoromethylphenyl)-glycine amide[3]

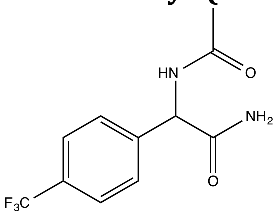

experimental data

Table S1.2.1: reaction conditions		
temperature T		37 °C
pH / pH* / pH** / pD		pH** 7.4[4]
ionic strength I / M		1.0
constant ionic strength		yes
solvent		aqueous buffers without co-solvent
process followed		H/D exchange
technique used		^{1}H NMR
relevant substrate pK_as		None relevant. Racemisation is presumed to proceed through the neutral form. Deprotonation of the amide is likely to be to a degree much less than 1%.
DOI		http://orca.cf.ac.uk/55130/1/U585519.pdf

Dependence of rate of deuteration upon [HPO_4^{2-}] indicates that for *rate* = $k_{2,\text{ neut, HPO4\_2-}}$ × [HPO_4^{2-}] × [neutral substrate]:

$k_{2,\text{ neut, HPO4\_2-}}$ **= 7.87 x 10^{-6} M^{-1}s^{-1}**

computed values

ΔΔG = -51.0 kcal/mol for:

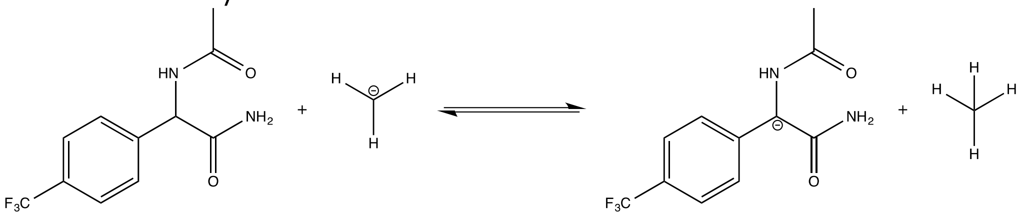

Sum of group contributions = -32.8 (primary amide) -8.9 (reverse secondary amide) -19.9 (benzene) +15.0 (cross-conjugation correction) = -46.6 kcal/mol

S1.3 N-Acetylthiophenylglycine methyl esters[3]

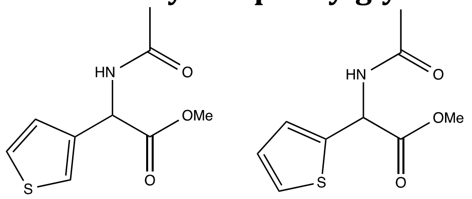

experimental data

**Table S1.3.1: reaction conditions**		
temperature T		37 °C
pH / pH* / pH** / pD		pH** 7.4[4]
ionic strength I / M		1.0
constant ionic strength		yes
solvent		aqueous buffers without co-solvent
process followed		H/D exchange
technique used		^{1}H NMR
relevant substrate pK_as		None relevant. Racemisation is presumed to proceed through the neutral form. Deprotonation of the amide is likely to be to a degree much less than 1%.
DOI		http://orca.cf.ac.uk/55130/1/U585519.pdf

Data were corrected for the simulataneous hydrolysis reaction as shown for the analogous esters in Section S1.1.

Dependence of rate of deuteration upon [HPO_4^{2-}] indicates that
for *rate* = $k_{2,\text{ neut, HPO4\_2-}}$ × [HPO_4^{2-}] × [neutral substrate]:

Thiophen-2-yl $k_{2,\text{ neut, HPO4\_2-}}$ = 2.74 x 10^{-3} $M^{-1}s^{-1}$
Thiophen-3-yl $k_{2,\text{ neut, HPO4\_2-}}$ = 5.62 x 10^{-5} $M^{-1}s^{-1}$

These rate constants are taken individually for the full QM calculations and plotted as a geometric mean for the group contribution method.

computed values
Thiophen-2-yl ΔΔG = -56.4 kcal/mol
Thiophen-3-yl ΔΔG = -52.2 kcal/mol

Sum of group contributions = -38.6 (ester) -8.9 (reverse secondary amide) -34.1 (five membered aromatic ring) +15.0 (cross-conjugation correction) = -66.6 kcal/mol

S1.4 5-Substituted hydantoins [5]

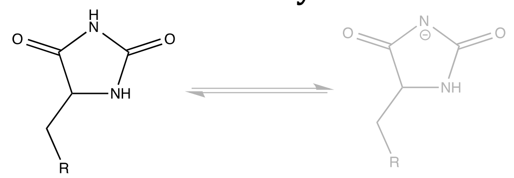

experimental data

Table S1.4.1: reaction conditions		
temperature T		25 °C
pH / pH* / pH** / pD		pH* 7.2
ionic strength I / M		1.0
constant ionic strength		Yes
solvent		aqueous buffers without co-solvent
process followed		H/D exchange
technique used		^{1}H NMR
relevant substrate pK_as		A pK_a of 8.65±0.10 has been reported by Lazarus, in agreement with the pK_a** of 8.65±0.06 reported by Narduolo. These are in reasonable agreement with the pK_a of 9.2 reported for 5-methylhydantoin).[6] Racemisation is presumed to proceed through the neutral form.
DOI		http://orca.cf.ac.uk/55132/1/U585521.pdf

Racemisation in water-DMSO mixture has been reported previously,[7] the values presented here are for aqueous solution.

For 5-benzylhydantoin (R=Ph), at 25°C and 0.5 M phosphate buffer k_{deut} = 5.42 x 10^{-6} s^{-1}
Activation enthalpy for 5-benzylhydantoin is 22.53±0.60 kcal/mol,[7] and hence E_a = 23.12 kcal/mol and hence $k(37^{\circ}C) \approx k(25^{\circ}C) \times e^{Ea(1/298-1/310)/R} = k(25^{\circ}C) \times 4.53$

k_2 = 4.91 x 10^{-5} $M^{-1}s^{-1}$ for Rate = k_2 × [total phosphate] × [Total 5-benzylhydantoin] at 37 °C

Correcting for the protonation state: at pH 7.2, 96.6 % of the hydantoin is neutral hence

$k_{2,neut}$ = 5.08 x 10^{-5} $M^{-1}s^{-1}$ corresponding to Rate = $k_{2,neut}$×[total phosphate]×[neutral hydantoin]

At pH 7.2, 71% of the buffer is in its basic form and therefore $\boldsymbol{k_{2,prot,HPO4\ 2-}}$ **= 7.16 x 10^{-5} $M^{-1}s^{-1}$** for rate = $k_{2,\ prot,\ HPO4\_2-}$×[HPO_4^{2-}]×[neutral hydantoin] corresponding to the reaction:

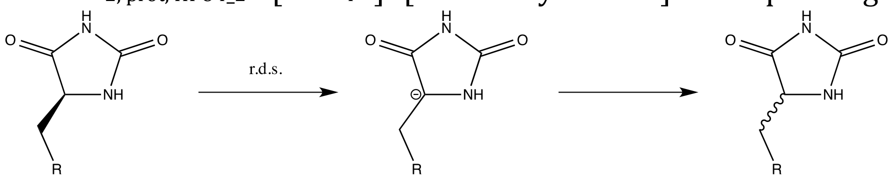

By analogy and using the same pK_a values for hydantoin and phosphate:

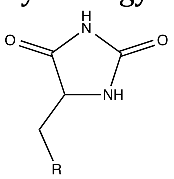

R = p-fluorophenyl $k_{2,neut}$ = 8.92 x 10^{-5} $M^{-1}s^{-1}$
R = p-methoxyphenyl $k_{2,neut}$ = 5.84 x 10^{-5} $M^{-1}s^{-1}$
R = 2-tetrahydrofuranyl $k_{2,neut}$ = 1.47 x 10^{-4} $M^{-1}s^{-1}$

R = OH $k_{2,neut}$ = 2.81 x 10^{-4} $M^{-1}s^{-1}$
R = m-pyridyl $k_{2,neut}$ = 1.23 x 10^{-4} $M^{-1}s^{-1}$
R = 2-N-methylpyrrolyl $k_{2,neut}$ = 7.92 x 10^{-5} $M^{-1}s^{-1}$

computed values

ΔΔG = -48.2 kcal/mol for:

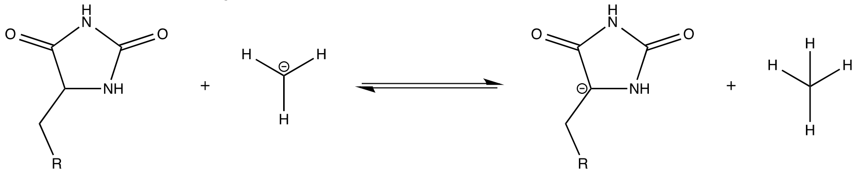

5-alkyl, 3-H hydantoins:
R = p-fluorophenyl ΔΔG = -48.9 kcal/mol
R = p-methoxyphenyl ΔΔG = -47.9 kcal/mol
R = 2-tetrahydrofuranyl ΔΔG = -48.0 kcal/mol
R = OH ΔΔG = -51.5 kcal/mol
R = m-pyridyl ΔΔG = -49.6 kcal/mol
R = 2-N-methylpyrrolyl ΔΔG = -47.0 kcal/mol

Sum of group contributions = -42.8 (acidic secondary amide, neutral) -8.9 (reverse secondary amide) +2.8 (alkyl) = -48.9 kcal/mol

S1.5 5-Substituted 3-*N*-methylhydantoins[5]

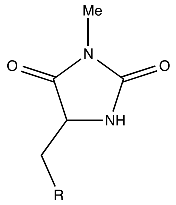

experimental data

Table S1.5.1: reaction conditions		
temperature T		25 °C
pH / pH* / pH** / pD		pH* 7.2
ionic strength I / M		1.0
constant ionic strength		Yes
solvent		aqueous buffers without co-solvent
process followed		H/D exchange
technique used		^{1}H NMR
relevant substrate pK_as		None relevant. Racemisation is presumed to proceed through the neutral form.
DOI		http://orca.cf.ac.uk/55132/1/U585521.pdf

By analogy with the 5-substituted hydantoins (S1.4) but without requiring a correction for the protonation state of the hydantoin (correction still included for fraction phosphate as HPO_4^{2-}):

R = phenyl k_2 = 4.30 x 10^{-5} M^{-1}s^{-1}
R = p-fluorophenyl k_2 = 3.89 x 10^{-5} M^{-1}s^{-1}
R = p-methoxyphenyl k_2 = 3.14 x 10^{-5} M^{-1}s^{-1}
R = m-pyridyl k_2 = 4.19 x 10^{-5} M^{-1}s^{-1}

computed values

ΔΔG = -48.2 kcal/mol for:

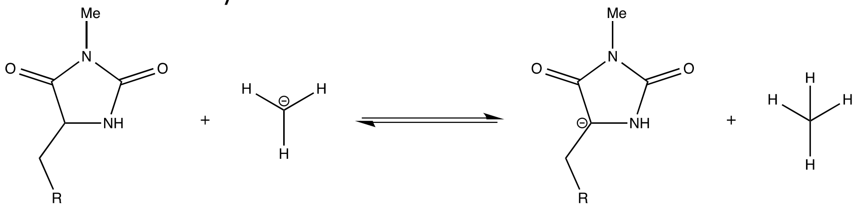

5-alkyl, 3-methyl hydantoins:
R = phenyl ΔΔG = -46.5 kcal/mol
R = p-fluorophenyl ΔΔG = -47.1 kcal/mol
R = p-methoxyphenyl ΔΔG = -45.5 kcal/mol
R = m-pyridyl ΔΔG = -47.6 kcal/mol

Sum of group contributions = -42.8 (acidic secondary amide, neutral) -8.9 (reverse secondary amide) +2.8 (alkyl) = -48.9 kcal/mol

S1.6 5-Phenylhydantoin[5]

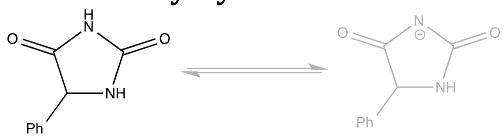

experimental data

Table S1.6.1: reaction conditions		
temperature T		25 °C
pH / pH* / pH** / pD		pH* 7.2
ionic strength I / M		1.0
constant ionic strength		Yes
solvent		aqueous buffers without co-solvent
process followed		H/D exchange
technique used		^{1}H NMR
relevant substrate pK_as		8.7 (assumed to be between that of hydantoin and 5,5-diphenylhydantoin).[6,8] Racemisation is presumed to proceed through the neutral form.
DOI		http://orca.cf.ac.uk/55132/1/U585521.pdf

Racemisation of 5-phenylhydantoin in water-DMSO mixture has been studied previously.[7] The results here are from our own studies in aqueous systems.

At 25°C and 0.1 M phosphate buffer k_{deut} = (408.0±13.2) x 10^{-6} s^{-1}

Activation enthalpy for 5-*benzyl*hydantoin is 22.53 kcal/mol and hence E_a = 23.12 kcal/mol and hence $k(37°C) \approx k(25°C) \times e^{Ea(1/298-1/310)/R} = k(25°C) \times 4.53$

$k_2 = 18.5\times10^{-3}\ M^{-1}s^{-1}$ for rate = k_2 × [total phosphate] × [Total 5-phenylhydantoin]

Correcting for the protonation state: at pH 7.2, 96.9 % of 5-phenylhydantoin is neutral hence $k_{2,neut} = 19.1\times10^{-3}\ M^{-1}s^{-1}$ corresponding to Rate = k_2×[total phosphate]×[neutral substrate]

At pH 7.2, 71% of the buffer is in its basic form and therefore $\boldsymbol{k_{2,prot,HPO4\ 2-} = 26.9\times10^{-3}\ M^{-1}s^{-1}}$ for rate = $k_{2,\ prot,\ HPO4\_2-}$×[HPO_4^{2-}]×[neutral hydantoin] corresponding to the reaction:

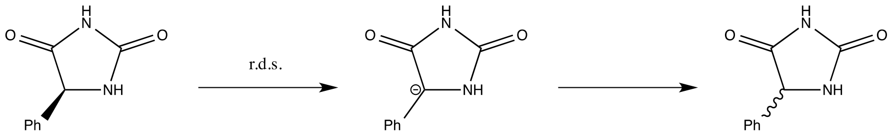

computed values

ΔΔG = -58.3 kcal/mol for:

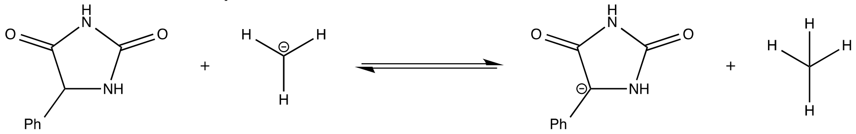

Sum of group contributions = -42.8 (acidic secondary amide, neutral) -8.9 (reverse secondary amide) -19.9 (phenyl) +15.0 (cross-conjugation correction) = -56.6 kcal/mol

S1.7 5-substituted 1-*N*-Acetyl-2-thiohydantoins[9]

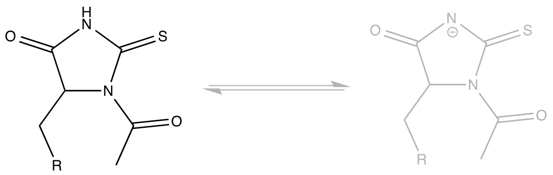

experimental data

Table S1.7.1: reaction conditions		
temperature T		37 °C
pH / pH* / pH** / pD		pH 7.4
ionic strength I / M		0.9
constant ionic strength		yes
solvent		aqueous buffers without co-solvent
process followed		Loss of ellipticity
technique used		CD
relevant substrate pK_as		6.95.[10] Racemisation is presumed to proceed through the neutral form.
DOI		http://orca.cf.ac.uk/78387/

The observed kinetics represent both H/D exchange and ester hydrolysis, as illustrated for compound **1a** in Scheme S1.7.1.

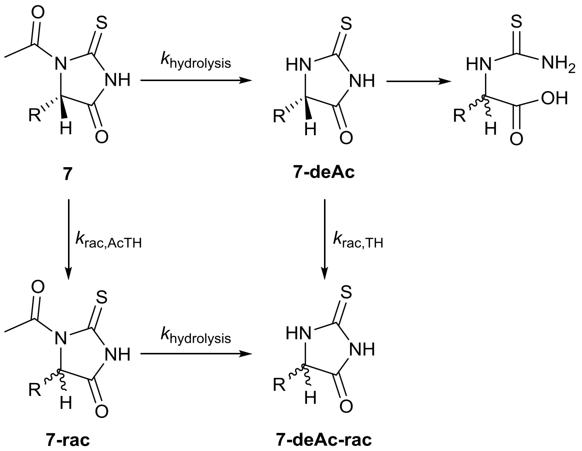

Scheme 1.7.1

The reactivity of **7** is studied using circular dichroism spectroscopy. Racemisation of **7-deAc** is much faster than racemization of **7** so that **7-deAc** makes a negligible contribution to the observed ellipticity. The observed rate constant for loss of ellipticity, k_θ, thus corresponds to $k_{rac,AcTH}$ + $k_{hydroysis}$. The hydrolysis of combined **7** and **7-rac** to form **7-deAc** and **7-deAc-rac** was followed using UV-visible spectroscopy; the subsequent hydrolysis of the thiohydantoin ring is slow on the timescale of loss of the acetyl group. This yields $k_{hydrolysis}$, allowing the required $k_{rac,AcTH}$ to be determined as $k_\theta - k_{hydrolysis}$.

For 1-acetyl-5-methyl-2-thiohydantoin at 37°C
$k_2 = 735 \times 10^{-6}$ M^{-1}s^{-1} for rate = k_2×[HPO_4^{2-}]×[Total 1-Acetyl-5-methyl-2-thiohydantoin]

Correcting for the protonation state: at pH 7.4, 26.19 % of the thiohydantoin is neutral hence $\mathbf{k_{2,neut,HPO4\,2-}}$ **= 2.81 x 10^{-3} M^{-1}s^{-1}** for rate = $k_{2,\ neut,\ HPO4\_2-}$×[HPO_4^{2-}]×[Neutral 1-Acetyl-5-methyl-2-thiohydantoin]

Analogously, and using the same pK_a value for the thiohydantoins,

For 1-acetyl-5-benzyl-2-thiohydantoin at 37°C, k_2 = 1347×10^{-6} $M^{-1}s^{-1}$ for rate = k_2×[HPO_4^{2-}]×[Total 1-Acetyl-5-benzyl-2-thiohydantoin]. Correcting for the protonation state: at pH 7.4, 26.19 % of the thiohydantoin is neutral hence $\boldsymbol{k_{2,neut,HPO4\ 2-}}$ **= 5.14 x 10^{-3} $M^{-1}s^{-1}$** for rate = $k_{2,\ neut,\ HPO4\_2-}$×[HPO_4^{2-}]×[Neutral 1-Acetyl-5-benzyl-2-thiohydantoin]

For 1-acetyl-5-(CH_2-indolyl)-2-thiohydantoin at 37°C, k_2 = 636×10^{-6} $M^{-1}s^{-1}$ for rate = k_2[HPO_4^{2-}][Total 1-Acetyl-5-(CH_2-indolyl)-2-thiohydantoin]. Correcting for the protonation state: at pH 7.4, 26.19 % of the thiohydantoin is neutral hence $\boldsymbol{k_{2,neut,HPO4\ 2-}}$ **= 2.43 x 10^{-3} $M^{-1}s^{-1}$** for rate = $k_{2,\ neut,\ HPO4\_2-}$×[HPO_4^{2-}]×[Neutral 1-Acetyl-5-(CH_2-indolyl)-2-thiohydantoin]

For 1-acetyl-5-(CH_2CH_2SMe)-2-thiohydantoin at 37°C, k_2 = 2465×10^{-6} $M^{-1}s^{-1}$ for rate = k_2[HPO_4^{2-}][Total 1-Acetyl-5-(CH_2CH_2SMe)-2-thiohydantoin]. Correcting for the protonation state: at pH 7.4, 26.19 % of the thiohydantoin is neutral hence $\boldsymbol{k_{2,neut,HPO4\ 2-}}$ **= 9.41 x 10^{-3} $M^{-1}s^{-1}$** for rate = $k_{2,\ neut,\ HPO4\_2-}$×[HPO_4^{2-}]×[Neutral 1-Acetyl-5-(CH_2CH_2SMe)-2-thiohydantoin]

computed values

For:

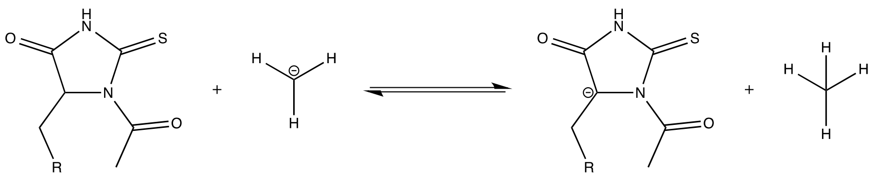

R = H ΔΔG = -58.7 kcal/mol

R = phenyl ΔΔG = -57.8 kcal/mol

R = indolyl ΔΔG = = -56.9 kcal/mol

R = CH_2SMe ΔΔG = -57.2 kcal/mol

Sum of group contributions = -16.2 (aminothio,oxo imide) -42.8 (acidic secondary amide, neutral) +2.8 (alkyl) = -56.2 kcal/mol

S1.8 3-*N*-phenyl-5-benzyl-2-thiohydantoin[11]

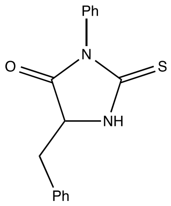

experimental data

Table S1.8.1: reaction conditions		
temperature T		37 °C
pH / pH* / pH** / pD		7.4
ionic strength I / M		0.9
constant ionic strength		yes
solvent		aqueous buffers without co-solvent
process followed		Loss of ellipticity
technique used		CD
relevant substrate pK_as		No relevant pK_as. Racemisation is presumed to proceed through the neutral form.
DOI		http://orca.cf.ac.uk/78387/

At 37°C
k_2 = 7.66 × 10^{-2} $M^{-1}s^{-1}$
Rate = k_2×[HPO_4^{2-}]×[Total 3-phenyl-5-benzyl-2-thiohydantoin]

No relevant protonation issues.
$k_{2,neut,HPO4\ 2-}$ = 7.66 x 10^{-2} $M^{-1}s^{-1}$ for rate = $k_{2,\ neut,\ HPO4\_2-}$×[HPO_4^{2-}]×[Neutral 3-phenyl-5-benzyl-2-thiohydantoin]

computed values
ΔΔG = -59.7 kcal/mol for:

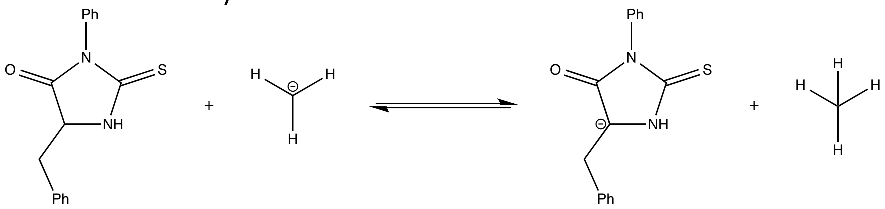

Sum of group contributions = -22.6 (reverse secondary thioamide) -42.8 (acidic secondary amide, neutral) +2.8 (alkyl) = -62.6 kcal/mol

S2 Second-order rate constants for phosphate-catalysed racemization reactions (literature data)

S2.9 Amfepramone[12]

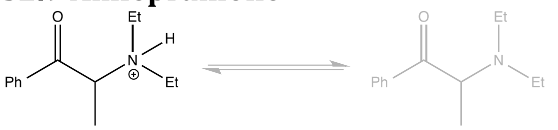

experimental data

Table S2.9.1: reaction conditions	
temperature T	37 °C
pH / pH* / pH** / pD	pD 7.4
ionic strength I	0.43 M
constant ionic strength	Yes
Solvent	aqueous buffers without co-solvent
process followed	H/D exchange
technique used	^{1}H-NMR spectroscopy
relevant substrate pK_as	8.79,[12] racemisation is presumed to proceed through the protonated form.
DOI	10.1002/chir.530070613

Reported dependence on [phosphate] (assumed to be $[\text{phosphate}]_{tot}$) of the rate constant for deuteration under the reaction conditions:

Table S2.9.2: reported rate constants

[phosphate]/M	k_{deut}/h^{-1}
6.70E-04	5.4E-04
1.30E-03	9.8E-03
2.00E-03	1.2E-02
1.30E-02	0.110
6.70E-02	0.58
0.133	1.28
0.2	2.33

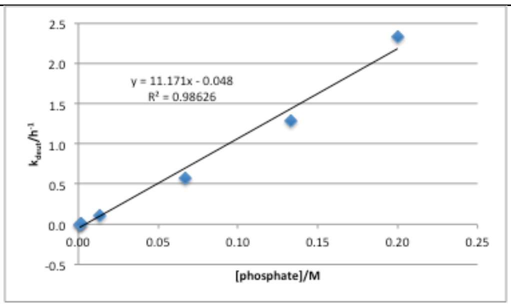

From the slope, k_2 = 3.1 x 10^{-3} M^{-1}s^{-1} for rate = k_2×[total buffer]×[total amfepramone] (Cf. estimated 3.0 x 10^{-4} M^{-1}s^{-1} at 25 °C from Mey et al.[13])

Correcting for the protonation state of amfepramone: at pH 7.4, 96.1 % is protonated hence $k_{2,prot}$ = 3.23 x 10^{-3} M^{-1}s^{-1} for rate = $k_{2,prot}$×[total buffer]×[protonated amfepramone]
At pH 7.4, 80 % of the buffer is in its basic form and therefore $\boldsymbol{k_{2,prot,HPO4\_2-}}$ **= 4.06 x 10^{-3} M^{-1}s^{-1}** for rate = $k_{2,\,prot,\,HPO4\_2-}$×[HPO_4^{2-}]×[protonated amfepramone] corresponding to the reaction:

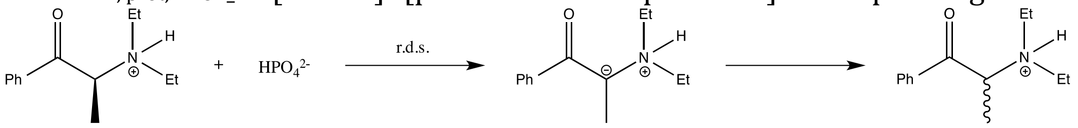

computed values

ΔΔG = -61.2 kcal/mol for:

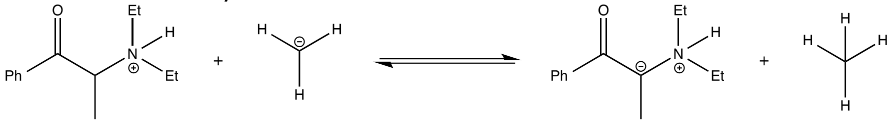

Sum of group contributions = -43.4 (ketone) -24.6 (dialkyl tertiary amine, protonated) + 2.8 (alkyl) = -65.2 kcal/mol

S2.10 Cathinone [12]

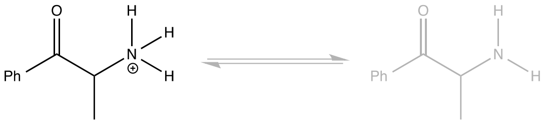

experimental data

**Table S2.10.1: reaction conditions**	
temperature T	37 °C
pH / pH* / pH** / pD	pD 7.4
ionic strength I / M	0.43
constant ionic strength	Yes
solvent	aqueous buffers without co-solvent
process followed	H/D exchange
technique used	^{1}H-NMR spectroscopy
relevant substrate pK_as	8.37,[12] racemisation is presumed to proceed through the protonated form.
DOI	10.1002/chir.530070613

Reported dependence on [phosphate] (assumed to be $[\text{phosphate}]_{tot}$) of the rate constant for deuteration under the reported conditions:

Table S2.10.2: reported rate constants

[phosphate]/M	k_{deut}/h^{-1}
6.70E-02	0.169
0.1	0.234
0.133	0.292
0.167	0.352
0.2	0.469

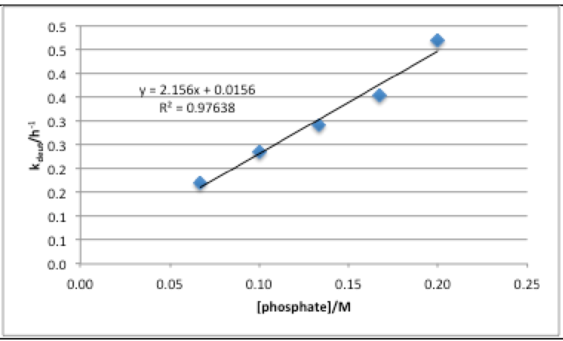

From slope, k_2 = 6.0 x 10^{-4} M^{-1}s^{-1} for rate = k_2×[total buffer]×[total cathinone]

Correcting for the protonation state of cathinone: at pH 7.4, 90.3 % is protonated hence

$k_{2,prot}$ = 6.64 x 10^{-4} M^{-1}s^{-1} for rate = $k_{2,prot}$×[total buffer]×[protonated cathinone]

At pH 7.4, 80% of the buffer is in its basic form and therefore $\boldsymbol{k_{2,prot,HPO4\_2-}}$ **= 8.35 x 10^{-4} M^{-1}s^{-1}** for rate = $k_{2,\text{ prot, HPO4\_2-}}$×[$HPO_4^{2-}$]×[protonated cathinone] corresponding to the reaction:

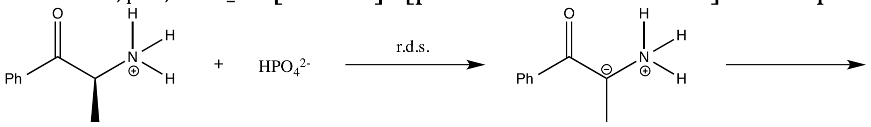

computed values

ΔΔG = -54.4 kcal/mol for:

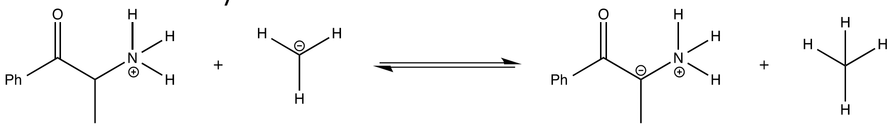

Sum of group contributions = -43.4 (ketone) -22.1 (primary amine, protonated) + 2.8 (alkyl) = -62.7 kcal/mol.

S2.11 Ketorolac [14]

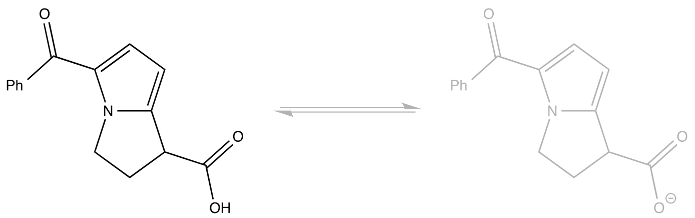

experimental data

Table S2.11.1: reaction conditions		
temperature T		25 ˚C
pH / pH* / pH** / pD		7.2
ionic strength I / M		Not discussed
constant ionic strength		Not discussed
solvent		aqueous buffers without co-solvent (<1 % DMSO)
process followed		Racemization
technique used		Chiral derivatization
relevant substrate pK_as		3.5 [14] Racemisation is presumed to proceed through the neutral form.
DOI		DOI: 10.1002/jps.2600840903

Reported dependence on [phosphate] of rate of racemization at pH=7.2 and 25°C:

Table S2.11.2: reported rate constants		
[phosphate]/M	k_{rac}/h^{-1}	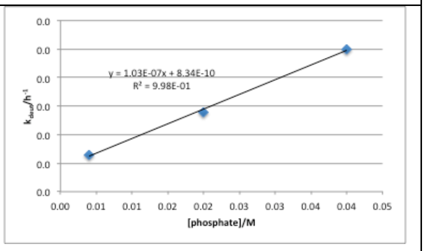
0.04	5.00 x 10^{-9}	
0.02	2.80 x 10^{-9}	
0.004	1.30 x 10^{-9}	

From slope, k_2 = 1.03 x 10^{-7} M^{-1}s^{-1}
Rate = k_2[GB][Total ketorolac]

Correcting for the protonation state: at pH 7.2, 0.02 % is neutral hence

$k_{2,neut}$ = 5.16 x 10^{-4} M^{-1}s^{-1} corresponding to Rate = $k_{2,neut}$×[total phosphate]×[neutral ketorolac]

At pH 7.2, 71% of the buffer is in its basic form and therefore **$k_{2,neut,HPO4\ 2-}$ = 7.27 x 10^{-4} M^{-1}s^{-1}** for rate = $k_{2,\ neut\ HPO4\_2-}$×[HPO_4^{2-}]×[neutral ketorolac] corresponding to the reaction:

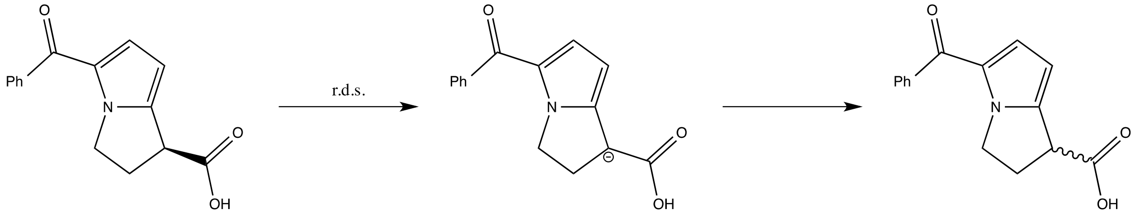

The measurements are for 25 ˚C and will be faster at 37 ˚C, reflected by an error bar representing 5-fold on the plot in the main text.

computed values

ΔΔG = -59.3 kcal/mol for:

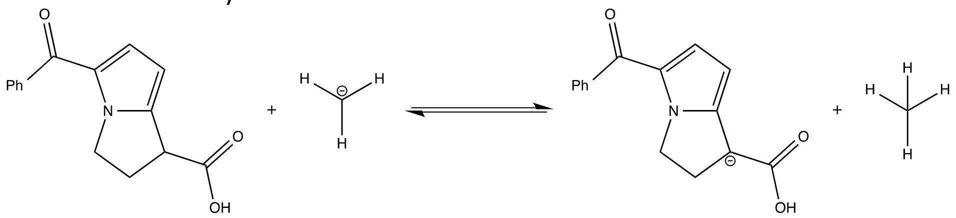

Sum of group contributions = -39.9 (carboxylic acid protonated form) -34.1 (five-membered aromatic ring) + 2.8 (alkyl) +15.0 (cross-conjugation correction) = -56.2 kcal/mol

S2.12 Pioglitazone (or Rosiglitazone)[15]

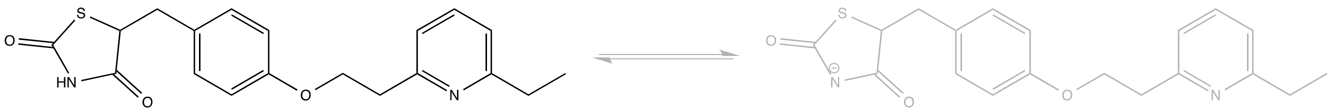

experimental data

Table S2.12.1: reaction conditions		
temperature T		37 ˚C
pH / pH* / pH** / pD		7.4
ionic strength I / M		Not specified
constant ionic strength		Not specified
solvent		aqueous buffers without co-solvent
process followed		Racemization
technique used		Chiral HPLC
relevant substrate pK_as		6.24[16] for triazolinedione NH. Racemisation is presumed to proceed through the neutral form.
DOI		10.1016/j.jpba.2007.09.004

Reported half life for racemization at pH 7.4 at 37 ˚C and 25 mM phosphate buffer is 4 hours.

k_2 = 1.93 x 10^{-3} $M^{-1}s^{-1}$

Correcting for the protonation state: at pH 7.4, 6.47 % is neutral hence

$k_{2,neut}$ = 3.00 x 10^{-2} $M^{-1}s^{-1}$ corresponding to Rate = k_2×[total phosphate]×[neutral pioglitazine]

At pH 7.4, 80% of the buffer is in its basic form and therefore $\boldsymbol{k_{2,neut,HPO4\ 2-}}$ **= 3.72 x 10^{-2} $M^{-1}s^{-1}$** for rate = $k_{2,\ neut,\ HPO4\_2-}$×[HPO_4^{2-}]×[neutral pioglitazone]

computed values

Computed values for model system shown:

ΔΔG = -57.3 kcal/mol

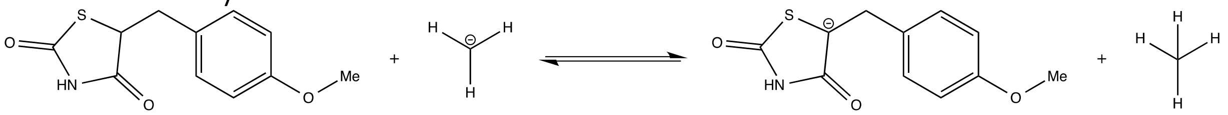

Sum of group contributions = -42.8 (secondary amide potentially acidic protonated form) -20.0 (thioether) +2.8 (alkyl) = -60.0 kcal/mol

S2.13 Thalidomide[17]

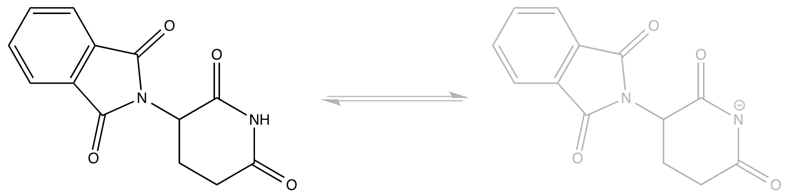

experimental data

Table S2.13.1: reaction conditions		
temperature T		37 °C
pH / pH* / pH** / pD		7.4
ionic strength I / M		0.8
constant ionic strength		Yes
solvent		aqueous buffers without co-solvent
process followed		Racemization
technique used		Chiral HPLC
relevant substrate pK_as		10.3[18] Racemisation is presumed to proceed through the neutral form.
DOI		10.1021/tx9801817

Reported dependence on [phosphate] gives k_2 = 0.24 h^{-1} at 37°C and pH 7.4.
where Rate = k_2[GB][Total thalidomide]

Correcting for the protonation state: at pH 7.4, 99.9 % is neutral hence

$k_{2,neut}$ = 6.67 x 10^{-5} M^{-1}s^{-1} corresponding to Rate = k_2×[total phosphate]×[neutral thalidomide]

At pH 7.4, 80% of the buffer is in its basic form and therefore **$k_{2,neut,HPO4\ 2-}$ = 8.34 x 10^{-5} M^{-1}s^{-1}** for rate = $k_{2,\ neut,\ HPO4\_2-}$×[HPO_4^{2-}]×[neutral thalidomide] corresponding to the reaction:

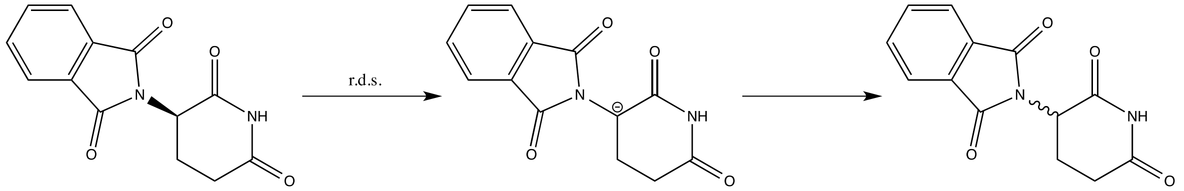

computed values

ΔΔG = -53.5 kcal/mol for:

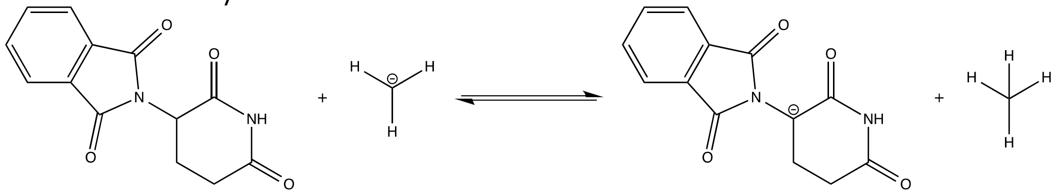

Sum of group contributions = -42.8 (potentially acidic secondary amide protonated form) -12.8 (imide) + 2.8 (alkyl) = -52.8 kcal/mol

S2.14 5-Methylhydantoin[7]

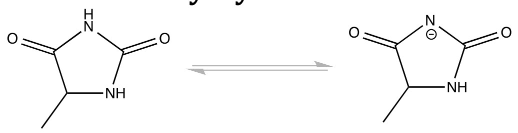

experimental data

Table S2.14.1: reaction conditions		
temperature T		37 °C
pH / pH* / pH** / pD		7.4
ionic strength I / M		1.1
constant ionic strength		Yes
solvent		1:1 phosphate buffer: DMSO
process followed		H/D exchange
technique used		^{1}H NMR
relevant substrate pK_as		9.2 by comparison to hydantoin and 5,5-dimethylhydantoin[6] Racemisation is presumed to proceed through the neutral form.
DOI		10.1002/hlca.19960790319

Studies in Water:DMSO 1:1 (compare to remaining hydantoins)

Reported k_2 = 0.052 h^{-1} at pD 7.4 and 37°C
Rate = k_2×[total phosphate]×[neutral methylhydantoin]

Correcting for the protonation state: at pH 7.2, 99.0 % is neutral hence $k_{2,neut}$ = 1.47 x 10^{-5} M^{-1}s^{-1}

At pH 7.4, 80 % of the buffer is in its basic form and therefore $\boldsymbol{k_{2,\mathrm{neutral,HPO4\ 2-}}}$ **= 1.83 x 10^{-5} M^{-1}s^{-1}** for rate = $k_{2,\mathrm{\ neutral,\ HPO4\_2-}}$×[$HPO_4^{2-}$]×[neutral methylhydantoin] corresponding to the reaction:

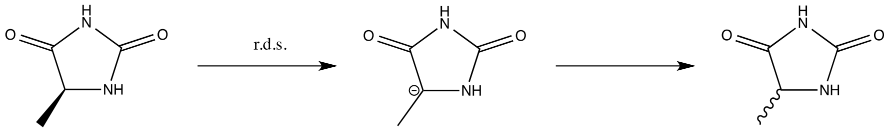

Uncertainty in the pK_a (measurements in 1:1 water:DMSO but pK_a for aqueous) are likely to be of the order of 0.5 pK_a units.

computed values

ΔΔG = -47.5 kcal/mol for:

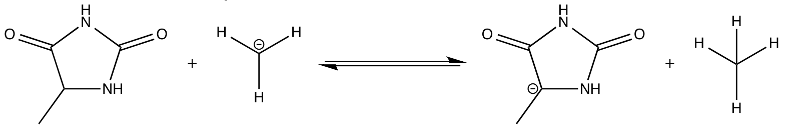

Sum of group contributions = -42.8 (acidic secondary amide, neutral) -8.9 (reverse secondary amide) +2.8 (alkyl) = -48.9 kcal/mol

S2.15 3-methyl-6-[1-(imidazol-1-yl)-1-(4-cyanophenyl)methyl] benzothiazolinone[19]

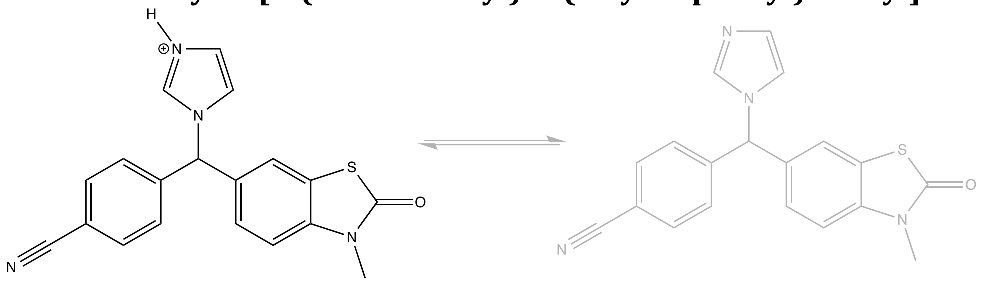

experimental data

Table S2.15.1: reaction conditions		
temperature T		37 ˚C
pH / pH* / pH** / pD		7.4
ionic strength I / M		Not discussed
constant ionic strength		Not discussed
solvent		aqueous buffers without co-solvent
process followed		Racemisation
technique used		Chiral capillary electrokinetic chromatography using highly sulfated cyclodextrins
relevant substrate pK_as		6.4 (approximated from pK_as for N-methylimidazole 7.4 and 1-benzylimidazole 6.7; assumed that adding a second aromatic group causes half as much change as the first i.e. 0.35 units hence pK_a =6.35)[20] Racemisation is presumed to proceed through the protonated form.
DOI		doi:10.1016/j.tetasy.2006.08.014

Reported $t_{1/2}$ at 37 ˚C and 0.05M phosphate buffer is 1988 hours, hence k_{rac} = 9.7 x 10^{-8} s^{-1} and k_2 = 1.94 x 10^{-6} M^{-1}s^{-1}

Correcting for the protonation state: at pH 7.4, 9.09 % is protonated hence $k_{2,prot}$ = 2.13 x 10^{-5} M^{-1}s^{-1} corresponding to Rate = $k_{2,prot}$×[total phosphate]×[protonated substrate]

At pH 7.4, 80% of the buffer is in its basic form and therefore **$k_{2,prot,HPO4\ 2-}$ = 3.33x 10^{-5} M^{-1}s^{-1}** for rate = $k_{2,\ prot,\ HPO4\_2-}$×[HPO_4^{2-}]×[protonated substrate] corresponding to the reaction:

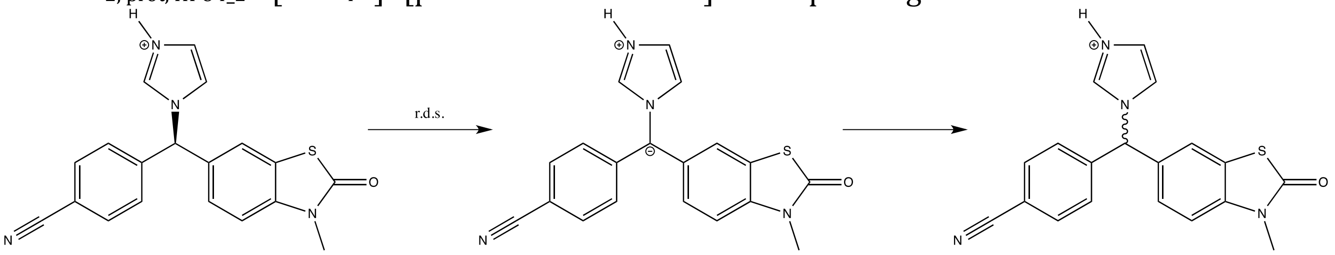

computed values

ΔΔG = -52.8 kcal/mol for:

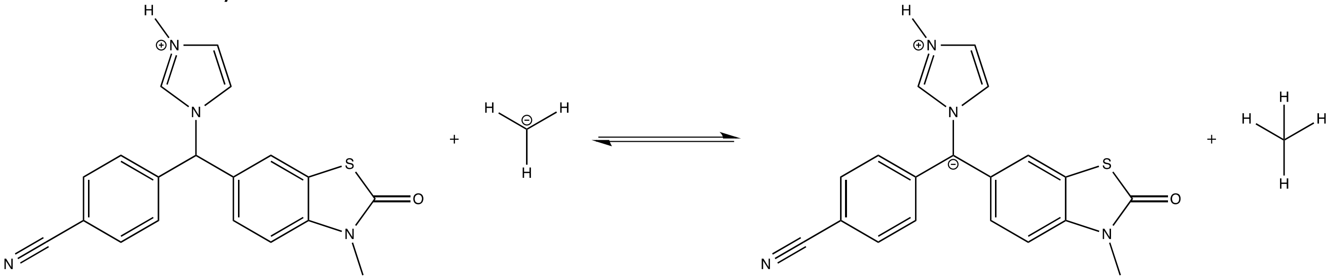

Sum of group contributions = -19.9 (benzene) -19.9 (benzene) -25.8 (protonated five-membered aromatic ring) +15.0 (cross-conjugation correction) = -50.6 kcal/mol

S2.16 Clopidogrel[21]

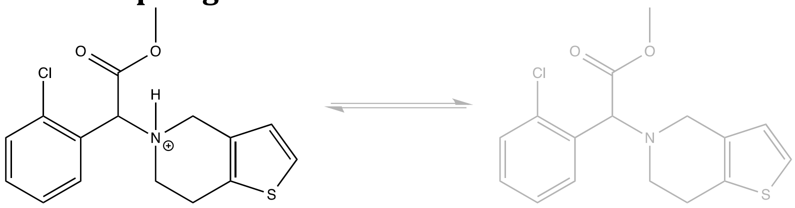

experimental data

Table S2.16.1: reaction conditions		
temperature T		37 °C
pH / pH* / pH** / pD		7.4
ionic strength I / M		0.78
constant ionic strength		yes
solvent		1:1 MeOH-water
process followed		racemisation
technique used		chiral HPLC
relevant substrate pK_as		Aqueous 4.55[22] (*Cf.* prasugrel 5.1[23]), racemisation is presumed to proceed through the protonated form. Amine bases in 1:1 MeOH-water mixtures are less basic by 0.48 pK_a units.[24] Hence, pK_a is 4.07.
reference		"Very Slow Chiral Inversion of Clopidogrel in Rats: A Pharmacokinetic and Mechanistic Investigation", Drug Metab. Dispos., 2000; 28(12), p.1405-1410.

Reported dependence on [phosphate] (assumed to be [phosphate]$_{tot}$) of the rate constant for racemisation under the reported conditions:

Table S2.16.2: reported rate constants		
[phosphate]/M	k_{rac}/day^{-1}	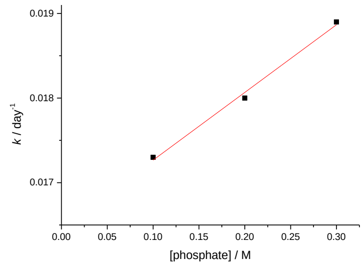
0.1	1.73×10^{-2}	
0.2	1.80×10^{-2}	
0.3	1.89×10^{-2}	

From slope k_2 = 9.26×10^{-8} M^{-1}s^{-1} for rate = k_2×[total buffer]×[total clopidogrel]

Correcting for the protonation state of clopidogrel: at pH 7.4, 0.047 % of the clopidogrel is protonated hence $k_{2,prot}$ = 1.97×10^{-6} M^{-1}s^{-1} for rate = k_2×[total buffer]×[protonated clopidogrel]

At pH 7.4, 80 % of the buffer is in its basic form and therefore $\boldsymbol{k_{2,prot,HPO4\ 2-}}$ **= 2.46 x 10^{-6} M^{-1}s^{-1}** for rate = $k_{2,\ prot,\ HPO4\text{-}2\text{-}}$×[$HPO_4^{2-}$]×[protonated clopidogrel] corresponding to the reaction:

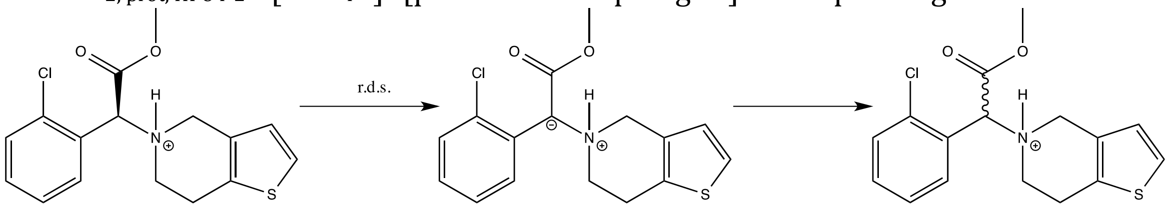

The uncertainty in this value is large. The uncorrected value of k_2 appears reasonable; the original publication corrects this value to 0% methanol as rate of deuteration at pD=7.4 and 37°C and 0.1 M phosphate buffer = 0.05 day^{-1}. Assuming no uncatalysed reaction (contrary to what Table S2.16.2 suggests), this corresponds to $k_2 = 5.78 \times 10^{-6}$ $M^{-1}s^{-1}$. When corrected using the aqueous pK_a , this gives $k_{2,prot} = 1.23\times10^{-4}$ $M^{-1}s^{-1}$ for rate = $k_{2,prot}\times$[total buffer]×[protonated clopidogrel] and $k_{2,prot,HPO4\_2-} = 1.54 \times 10^{-4}$ $M^{-1}s^{-1}$ for rate = $k_{2,\,prot,\,HPO4\_2-}\times[HPO_4^{2-}]\times$[protonated clopidogrel]. This is approximately 60 fold larger than the rate constant estimated above. Uncertainty arises from the experimental pK_a which comes from material provided without experimental details by the drug's manufacturers. The pK_a for a related compound is available and is higher but assignment is not straightforward. Although it is not a substantial outlier, we consider that this point is only assignable to within an order of magnitude and involves a number of assumptions that can place it at this point or else substantially below the line, this is reflected on the chart in the main text and the point is excluded from the fitting.

computed values

ΔΔG = -62.9 kcal/mol for:

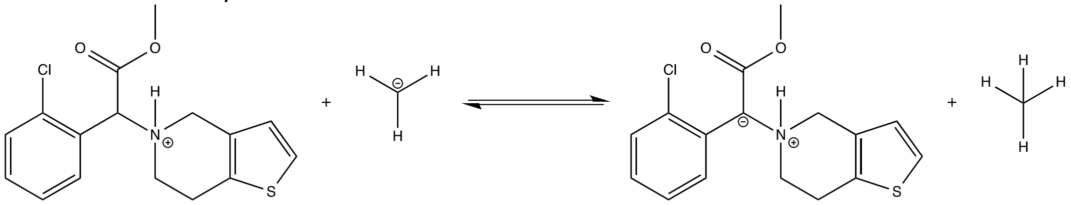

Sum of group contributions = -38.6 (ester) -24.6 (dialkyl tertiary amine, protonated) -19.9 (phenyl) +15.0 (cross-conjugation correction) = -68.1 kcal/mol

S3. Hammett plots for compounds 1a-h.

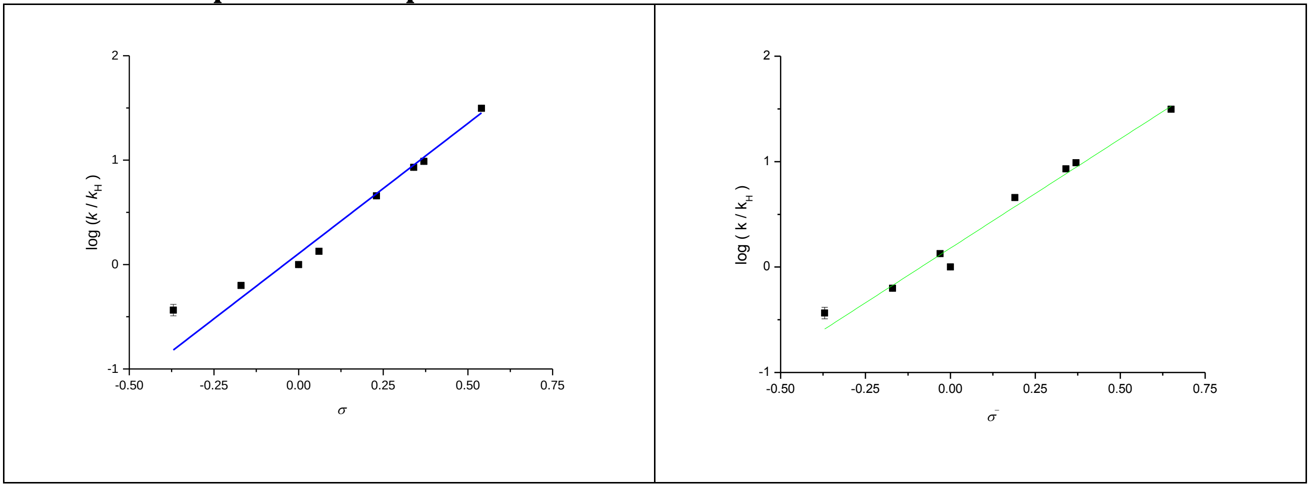

Figure S1: Hammett plots for the general base catalysed H/D exchange at the stereogenic centres of **1a-h**, in D_2O phosphate buffers of *I* = 1 M at 37 °C as a function of σ (*Left*) and as a function of σ^- (*Right*); Lines are lines of best linear fit.

The absence of clear breaks from linearity of both plots in Figure S1 suggests a common mechanism of H/D exchange for compounds **1a-h**. The positive gradients seen in Figure S1 suggest that negative charge is built up on the reaction centre during H/D exchange. The kinetic data show a reasonable linear correlation with the Hammett σ parameter, yielding a Hammett ρ of 2.50 ± 0.03 with a corresponding R^2 of 0.979 but Figure S1 shows a better overall correlation of the kinetic data with σ^- (ρ = 2.07 ± 0.02, R^2 = 0.984).

S4. Relationship between log(k_{gb}) and ΔG(R_1,R_2,R_3) for compounds 1-8.

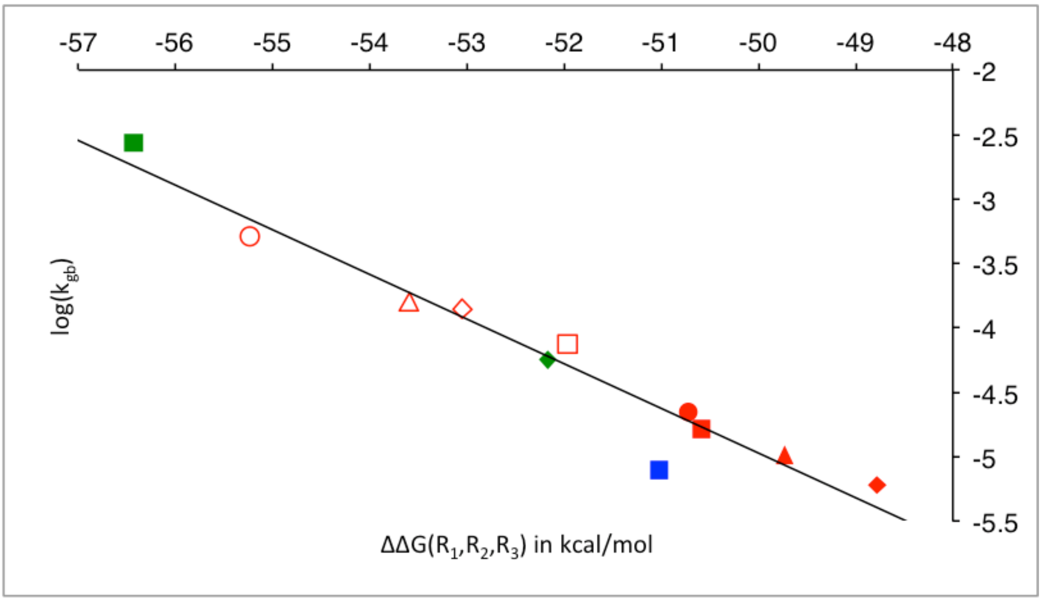

Figure S2. Second-order rate constants for general-base-catalyzed racemisation plotted against computed deprotonation free energies for arylglycine esters: **1a** (■), **1b** (◆), **1c** (▲), **1d** (●), **1e** (□), **1f** (◇), **1g**(△), **1h** (○),**3a** (■) and **3b** (◆) and arylglycine amide **2** (■).

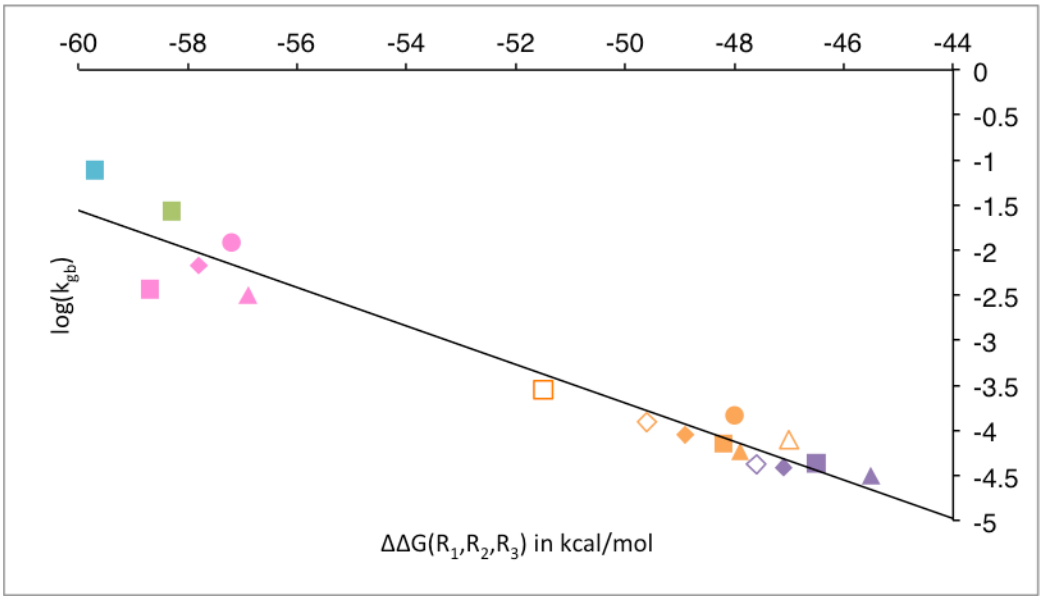

Figure S3. Second-order rate constants for general-base-catalyzed racemisation plotted against computed deprotonation free energies for hydantoins and thiohydantoins: **4a** (■), **4b** (◆), **4c** (▲), **4d** (●) **4e** (□), **4f** (◇), **4g** (△), **5a** (■), **5b** (◆), **5c** (▲), **5f** (◇), **6** (■), **7a** (■), **7b** (◆), **7c** (▲), **7d** (●) and **8** (■).

The compounds in Figure S2 yield a line of best fit with equation $\log(k_{gb}) = -0.35\times\Delta\Delta G(R_1,R_2,R_3) - 22.30$ and an R^2 value of 0.95. The compounds in Figure S3 fit a line of equation $\log(k_{gb}) = -0.21\times\Delta\Delta G(R_1,R_2,R_3) - 14.38$ with an R^2 value of 0.94.

S5. Example application of the group contribution method

The group contribution approach is illustrated with the example of compounds **9** and **15** (Figure S4). The three groups around the chiral centre (in addition to the hydrogen) are classified using the structural definitions provided in Section S7 (detailed definitions are provided in computer-readable SMARTS format along with human-readable names). For **9**, these are found to be ketone, dialkyl tertiary amine and alkyl. At neutral pH, the most prevalent form will be that in which the amine is protonated and this will also be the form that is most likely to racemise fastest. Therefore the appropriate contributions (taken from Section S8) are -43.4 for ketone, -24.6 for a protonated dialkyl tertiary amine and +2.8 for an alkyl group. There is no cross-conjugation correction in this case. This gives an overall value of -65.2 kcal/mol. Given the equation $\log(k_{gb}) = -0.11\times \Sigma\Delta\Delta G - 9.81$ for a non-aromatic anion, k_{gb} is expected to be 2.3×10^{-3} $M^{-1}s^{-1}$. For **15**, the groups are found to be benzene, benzene and protonated five-membered aromatic ring. The appropriate contributions (taken from Section S8) are -19.9 for benzene and -25.8 for a protonated five-membered aromatic ring. The cross-conjugation correction is +15 kcal/mol this case. This gives an overall value of -50.6 kcal/mol and hence k_{gb} of 5.7×10^{-5} $M^{-1}s^{-1}$. The alternative equation $\log(k_{gb}) = -0.26\times \Sigma\Delta\Delta G - 16.95$ can be used if an aromatic anion is suspected.

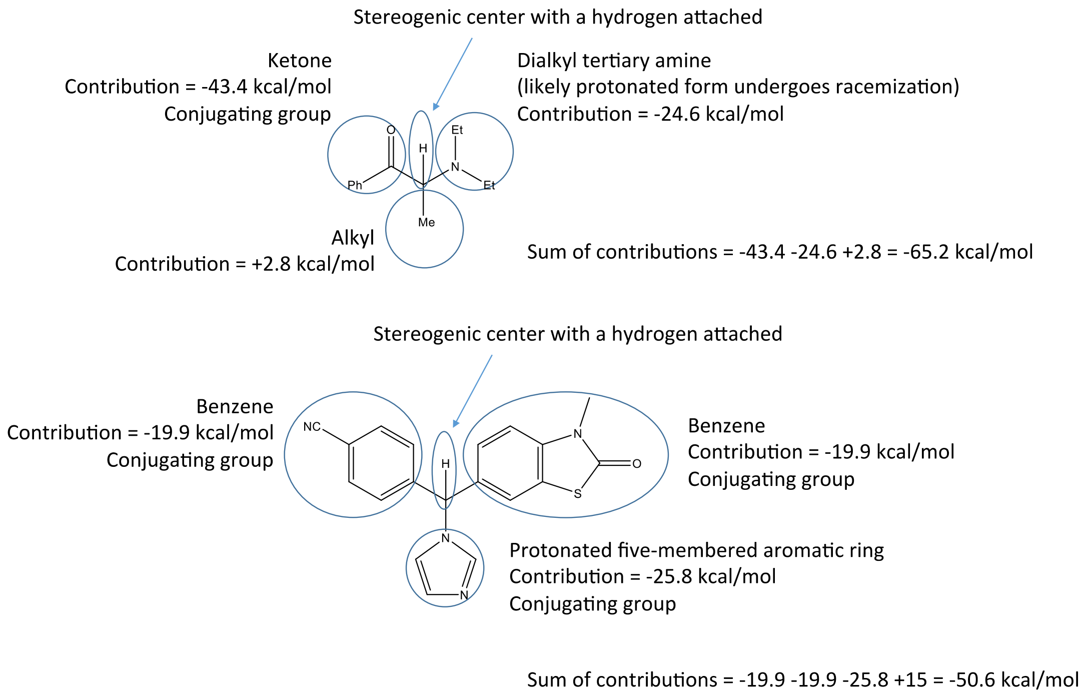

Figure S4. The group contribution approach illustrated for compounds **9** and **15**.

S6. Program for identifying and flagging groups attached to a chiral center (and adding [Xe] at the point of attachment to the chiral center)

```
//
// file chiral_chopper.cc
// David Cosgrove
//
// reads from stdin, writes to stdout.  Takes all chiral atoms, chops them out,
// mends the ends with Xe atoms and writes the new SMILES string out.
//
/*

This program is distributed under the following license:

Copyright (C) 2015
AstraZeneca, David Cosgrove

All rights reserved.

Redistribution and use in source and binary forms, with or without
modification, are permitted provided that the following conditions are
met:

   * Redistributions of source code must retain the above copyright
     notice, this list of conditions and the following disclaimer.
   * Redistributions in binary form must reproduce the above
     copyright notice, this list of conditions and the following
     disclaimer in the documentation and/or other materials provided
     with the distribution.
   * Neither the name of AstraZeneca nor the names of its
     contributors may be used to endorse or promote products derived
     from this software without specific prior written permission.

THIS SOFTWARE IS PROVIDED BY THE COPYRIGHT HOLDERS AND CONTRIBUTORS
"AS IS" AND ANY EXPRESS OR IMPLIED WARRANTIES, INCLUDING, BUT NOT
LIMITED TO, THE IMPLIED WARRANTIES OF MERCHANTABILITY AND FITNESS FOR
A PARTICULAR PURPOSE ARE DISCLAIMED. IN NO EVENT SHALL THE COPYRIGHT
OWNER OR CONTRIBUTORS BE LIABLE FOR ANY DIRECT, INDIRECT, INCIDENTAL,
SPECIAL, EXEMPLARY, OR CONSEQUENTIAL DAMAGES (INCLUDING, BUT NOT
LIMITED TO, PROCUREMENT OF SUBSTITUTE GOODS OR SERVICES; LOSS OF USE,
DATA, OR PROFITS; OR BUSINESS INTERRUPTION) HOWEVER CAUSED AND ON ANY
THEORY OF LIABILITY, WHETHER IN CONTRACT, STRICT LIABILITY, OR TORT
(INCLUDING NEGLIGENCE OR OTHERWISE) ARISING IN ANY WAY OUT OF THE USE
OF THIS SOFTWARE, EVEN IF ADVISED OF THE POSSIBILITY OF SUCH DAMAGE.

*/

#include <iostream>
#include <algorithm>

#include "oechem.h"
#include "oesystem.h"
```

```
using namespace std;
using namespace OEChem;
using namespace OESystem;

// **************************************************************************
// break all bonds to atom in mol, capping with Xe atoms. Note that a copy
// of original mol is used - we don't want to corrupt the original in case
// there is more than 1 chiral centre
void disconnect_chiral_atom( OEMol mol , OEAtomBase &atom ) {

 OEIter<OEAtomBase> cp_atom = mol.GetAtoms( OEHasAtomIdx( atom.GetIdx() ) );
 OEIter<OEBondBase> bond = cp_atom->GetBonds();
 for( ; bond ; ++bond ) {
   OEAtomBase *x_atom = bond->GetNbr( cp_atom );
   OEAtomBase *new_atom = mol.NewAtom( OEElemNo::Xe );
   mol.NewBond( x_atom , new_atom , bond->GetOrder() );
   mol.DeleteBond( bond );
 }
 mol.DeleteAtom( cp_atom );

 string new_smi;
 OECreateCanSmiString( new_smi , mol );

 cout << new_smi << " " << mol.GetTitle() << endl;

}

// **************************************************************************
int main( int argc , char **argv ) {

 oemolistream ims;
 OEMol mol;

 while( ims >> mol ) {
   OEAddExplicitHydrogens( mol );
   OEPerceiveChiral( mol );
   OEIter<OEAtomBase> atom = mol.GetAtoms( OEIsChiralAtom() );
   for( ; atom ; ++atom ) {
     if( OEElemNo::C != atom->GetAtomicNum() )
          continue;
     disconnect_chiral_atom( mol , atom );
   }
 }

 return 0;

}
```

S7. SMARTS strings defining groups attached to a chiral center and encoded with [Xe] as the attachment point to the chiral center (generated using the program above)

Group	SMARTS pattern	Conjugating group
THIOCARBONYL	[Xe]C=[SD1]	Y
NITRO	[Xe]N(=O)(=O)	Y
REV_AMIDE_DB	[Xe][$CARBONYL]N=[A,a]	Y
TRIAZINE	[Xe]c1ncncn1	Y
KETONE	[Xe]C(=O)[C,c]	Y
SEC_AMIDE_ACIDIC	[Xe]C(=O)[ND2][$CARBONYL,$AROMATIC_DEF]	Y
NITRILE	[Xe]C#N	Y
CARBOXYLIC_ACID	[Xe]C(=O)[OD1]	Y
PYRIMIDINE_2_4	[Xe]c1ncncc1	Y
ESTER	[Xe]C(=O)[OD2][A,a]	Y
PYRIDAZINE_3_4	[Xe]c1cnncc1	Y
TERT_AMIDE_DIAROMATIC	[Xe]C(=O)[ND3]([$AROMATIC_DEF])[$AROMATIC_DEF]	Y
HYDROXAMIC_ACID	[Xe]C(=O)[ND2][OD1]	Y
PYRAZINE	[Xe]c1nccnc1	Y
PYRIMIDINE_2_6	[Xe]c1ncccn1	Y
FIVE_MEM_AROMATIC	[Xe][$FIVE_MEMBERED_AROMATIC_DEF]	Y
TERT_AMIDE_OTHER	[Xe][$TERT_AROMATIC_AMIDE_DEF,$TERT_AMIDE_OTHER_DEF,$TERT_AMIDE_ALKYL_OTHER_DEF,$TERT_AMIDE_AROMATIC_OTHER_DEF]	Y
PYRIDAZINE_2_3	[Xe]c1nnccc1	Y
PRIM_AMIDE	[Xe]C(=O)[ND1]	Y
SIX_MEMBERED_GENERAL	[$SIX_MEMBERED_GENERAL_DEF;!$BENZENE;!$TRIAZINE;!$PYRADIZINE_3_4;!$PYRADIZINE_2_3;!$PYRAZINE;!$PYRIMIDINE_3_5;!$PYRIMIDINE_2_4;!$PYRIMIDINE_2_6;!$PYRIDINE_PARA;!$PYRIDINE_META;!$PYRIDINE_ORTH]	Y
TERT_AMIDE_ALKYL_AROMATIC	[Xe]C(=O)[ND3]([$ALKYL_DEF])[$AROMATIC_DEF]	Y
ALKYNE	[Xe]C#C	Y
PYRIDINE_PARA	[Xe]c1ccncc1	Y
TERT_AMIDE_DIALKYL	[Xe]C(=O)[ND3]([$ALKYL_DEF])[$ALKYL_DEF]	Y
SEC_AMIDE_NONACIDIC	[Xe]C(=O)[ND2][$ALKYL_DEF]	Y
SEC_AMIDE_OTHER	[Xe]C(=O)[ND2][!$SEC_AMIDE_OTHER_DEF]	Y
PYRIMIDINE_3_5	[Xe]c1cncnc1	Y
PYRIDINE_ORTHO	[Xe]c1ncccc1	Y
ISONITRILE	[Xe][$ISONITRILE_1DEF,$ISONITRILE_2DEF]	Y
REVERSE_IMINE	[Xe]C=[N+0]	N
QUAT_AMMONIUM	[Xe][NX4;!$QA_DEF]	N
PYRIDINE_META	[Xe]c1cnccc1	Y
ALKENE	[Xe]C=[C,c]	Y
THIOETHER	[Xe][SD2]-[A,a]	N
BENZENE	[Xe]c1ccccc1	Y
IMINE	[Xe][ND2]=[CX3,c]	Y
HALOGEN (Cl)	[Xe][Cl]	N
AMINOTHIO_OXO_IMIDE	[Xe][ND3]([$CARBONYL])[$THIOAMIDE]	N
IMIDE	[Xe][ND3]([$CARBONYL])[$CARBONYL]	N
EPOXIDE	[Xe][OD2][$C_NEXT_TO_CHIRAL]	N

REVERSE_ESTER	[Xe][OD2][$CARBONYL]	N
ETHER_DB	[Xe][OD2][$ALKENE_DEF,$ALK_ETHER_N_DEF,$ALK_ETHER_S_DEF]	N
HALOGEN (F)	[Xe][F]	N
REVERSE_TERT_AMIDE	[Xe][ND3]([A,a;!$CARBONYL])[$CARBONYL]	N
REVERSE_SEC_AMIDE	[Xe][ND2][$CARBONYL]	N
SEC_AMINE_ACIDIC	[Xe][ND2][$AROMATIC_DEF;!$CARBONYL]	N
AROMATIC_ETHER	[Xe][OD2][c]	N
TERT_AMINE_DIAROMATIC	[Xe][ND3]([$AROMATIC_DEF])[$AROMATIC_DEF]	N
TERT_AMINE_ALKYL_AROMATIC	[Xe][ND3]([$ALKYL_DEF])[$AROMATIC_DEF]	N
TERT_AMINE_OTHER	[$TERT_AMINE_OTHER_DEF,$TERT_AMINE_ALKYL_OTHER_DEF,$TERT_AMINE_AROMATIC_OTHER_DEF]	N
ETHER	[Xe][OD2][CX4;!$C_NEXT_TO_CHIRAL]	N
O_HYDROXYLAMINE	[Xe][OD2][N,n]	N
TERT_AMINE_DIALKYL	[Xe][ND3]([$ALKYL_DEF])[$ALKYL_DEF]	N
HYDROXYL	[Xe][OH1]	N
PROTON	[XeH1]	N
PRIM_AMINE	[Xe][ND1]	N
SEC_AMINE_NONACIDIC	[Xe][ND2][$ALKYL_DEF]	N
SEC_AMINE_OTHER	[Xe][ND2][!$AROMATIC_DEF;!$CARBONYL;!$ALKYL_DEF]	N
ALKYL	[Xe][CX4]	N

N.B. SMILES are first transformed into standard representations such as nitro to N(=O)=O.

SMARTS patterns used as recursive SMARTS in the table above:

ALKYL_DEF [CX4]
SIX_MEMBERED_GENERAL_DEF [Xe]a1aaaaa1
FIVE_MEMBERED_AROMATIC_DEF a1aaaa1
SIX_MEMBERED_AROMATIC_DEF a1aaaaa1
AROMATIC_DEF [$FIVE_MEMBERED_AROMATIC_DEF,$SIX_MEMBERED_AROMATIC_DEF]
CARBONYL C(=O)
C_NEXT_TO_CHIRAL C[Xe]
ALKENE_DEF C=C
ALK_ETHER_N_DEF C=N
ALK_ETHER_S_DEF C=S
SEC_AMIDE_OTHER_DEF [$CARBONYL,$AROMATIC_DEF,$ALKYL_DEF,OD1]
TERT_AMIDE_OTHER_DEF C(=O)[ND3]([!$AROMATIC_DEF;!$ALKYL_DEF])[!$AROMATIC_DEF;!$ALKYL_DEF]
TERT_AMIDE_ALKYL_OTHER_DEF C(=O)[ND3]([$ALKYL_DEF])[!$AROMATIC_DEF;!$ALKYL_DEF]
TERT_AMIDE_AROMATIC_OTHER_DEF C(=O)[ND3]([$AROMATIC_DEF])[!$AROMATIC_DEF;!$ALKYL_DEF]
TERT_AROMATIC_AMIDE_DEF C(=O)n
TERT_AMINE_OTHER_DEF
[Xe][ND3]([!$AROMATIC_DEF;!$ALKYL_DEF;!$CARBONYL])[!$AROMATIC_DEF;!$ALKYL_DEF;!$CARBONYL]
TERT_AMINE_ALKYL_OTHER_DEF [Xe][ND3]([$ALKYL_DEF])[!$AROMATIC_DEF;!$CARBONYL;!$ALKYL_DEF]
TERT_AMINE_AROMATIC_OTHER_DEF
[Xe][ND3]([$AROMATIC_DEF])[!$AROMATIC_DEF;!$ALKYL_DEF;!$CARBONYL]
QA_DEF [NH2;D2]=C
ISONITRILE_1DEF [N+]#C
ISONITRILE_2DEF N=[CD1;H0]
THIOAMIDE C(=S)N

ETHER_OTHER [Xe][OD2][!C;!c;!N;!n]
THIOL [Xe][SH1
SULFUR_OXIDE_DEF [Xe]S(=O
SEVEN_MEM_RING [Xe]a1aaaaaa1

FOUR_MEM_RING [Xe]a1aaa1
THREE_MEM_RING [Xe]a1aa1
IMINIUM [Xe]C=[N+1]
REV_IMINIUM [Xe][N+1]=C
SULFINIUM [Xe][SH1]=A
ASULFINIUM [Xe]C=[SH1;D2]
OXOIMINIUM [Xe]N(=O)=C
S_N_DB [Xe]S=N
PHOSPHOROUS_DERIV [Xe]P
ALDEHYDE [Xe][CD2]=O
ACID_CHLORIDE [Xe][$CARBONYL][Cl,F]
THIOESTER [Xe][$CARBONYL]S
NN_DB [Xe]N=[N+0]
N3 [Xe]N=[N+1]=[N-1]
N_C_OS [Xe]N=C=[O,S]
OTHER_ATOM [Xe][Se,Na,B,Sn,Si,As]
OTHER
[$OTHER_ATOM,$N3,$N_C_OS,$NN_DB,$THIOESTER,$ACID_CHLORIDE,$ALDEHYDE,$PHOSPHOROUS_DERIV,$THIOL,$ETHER_OTHER,$S_N_DB,$OXOIMINIUM,$ASULFINIUM,$SULFINIUM,$IMINIUM,$REV_IMINIUM,$THREE_MEM_RING,$FOUR_MEM_RING,$SULFUR_OXIDE_DEF,$SEVEN_MEM_RING]

S8. Quantum mechanical free energies for the representative example of each group type (structures provided as xyz file)

	Group	$G_{neutral}$	G_{anion}	Change to ΔG_{deprot}
1	THIOCARBONYL	-516.077005	-515.613546	-63.4
2	NITRO	-245.015871	-244.550548	-62.2
3	REV_AMIDE_DB	-247.262872	-246.77505	-48.1
4	TRIAZINE	-319.658918	-319.169703	-47.2
5	KETONE	-193.127433	-192.632202	-43.4
6<br>7	SEC_AMIDE_ACIDIC	-361.839631<br>(-361.368201)	-361.343346<br>(-360.844022)	-42.8<br>(-25.3)[a]
8	NITRILE	-132.752756	-132.253135	-40.7
9<br>10	CARBOXYLIC_ACID	-229.089451<br>(-228.6344)	-228.58857<br>(-228.094625)	-39.9<br>(-15.5)[a]
11	PYRIMIDINE_2_4	-303.604238	-303.102871	-39.6
12	ESTER	-268.35812	-267.855226	-38.6
13	PYRIDAZINE_3_4	-303.571456	-303.068284	-38.4
14	TERT_AMIDE_DIAROMATIC	-671.177519	-670.67282	-37.5
15<br>16	HYDROXAMIC_ACID	-284.37695<br>(-283.903281)	-283.871521<br>(-283.373566)	-37.0<br>(-21.8)[a]
17	PYRAZINE	-303.59581	-303.08642	-34.5
18	PYRIMIDINE_2_6	-303.606252	-303.096277	-34.2
19<br>20	FIVE_MEM_AROMATIC	-608.343159<br>(-265.941032)	-607.833031<br>(-265.417639)	-34.1<br>(-25.8)[b]
	TERT_AMIDE_OTHER	na	na	-33.4[h]
21	PYRIDAZINE_2_3	-303.571845	-303.060206	-33.1
22	PRIM_AMIDE	-209.211191	-208.699011	-32.8
	SIX_MEMBERED_GENERAL	na	na	-32.7[c]
23	TERT_AMIDE_ALKYL_AROMATIC	-479.474228	-478.960437	-31.8
24	ALKYNE	-116.640995	-116.127127	-31.7
25<br>26	PYRIDINE_PARA	-287.550678<br>(-287.998040)	-287.035746<br>(-287.520127)	-31.1<br>(-54.3)[b]
27	TERT_AMIDE_DIALKYL	-287.768711	-287.25375	-31.0
28	SEC_AMIDE_NONACIDIC	-248.493492	-247.976389	-29.7
	SEC_AMIDE_OTHER	na	na	-29.7[g]
29	PYRIMIDINE_3_5	-303.601827	-303.083519	-28.9
30<br>31	PYRIDINE_ORTHO	-287.551279<br>(-287.997482)	-287.031192<br>(-287.518454)	-27.8<br>(-53.6)[b]
32	ISONITRILE	-132.716115	-132.195923	-27.8
33	REVERSE_IMINE	-212.515321	-211.993469	-26.7
34	QUAT_AMMONIUM	-214.126098	-213.602286	-25.5
35	PYRIDINE_META	-287.549153	-287.023748	-24.5
36	ALKENE	-117.870653	-117.339407	-20.8
37	THIOETHER	-669.685155	-669.152543	-20.0
38	BENZENE	-271.498541	-270.965787	-19.9
39	IMINE	-212.51532	-211.981445	-19.2
40	HALOGEN (Cl)	-500.104783	-499.567730	-17.2
41	AMINOTHIO_OXO_IMIDE	-818.690216	-818.151667	-16.2
42	IMIDE	-401.109780	-400.565798	-12.8
43	EPOXIDE	-153.774298	-153.22966	-12.4
44	REVERSE_ESTER	-268.358289	-267.812737	-11.8

45	ETHER_DB	-209.158089	-208.611589	-11.3
46	HALOGEN (F)	-139.741591	-139.194597	-10.9
47	REVERSE_TERT_AMIDE	-287.768769	-287.218523	-8.9
48	REVERSE_SEC_AMIDE	-248.493503	-247.943211	-8.9
49<br>50	SEC_AMINE_ACIDIC	-663.685459<br>(-663.202251)	-663.134797<br>(-662.64163)	-8.6<br>(-2.4)[a]
51	AROMATIC_ETHER	-346.704069	-346.152173	-7.9
52	TERT_AMINE_DIAROMATIC	-557.819721	-557.266581	-7.1
53	TERT_AMINE_ALKYL_AROMATIC	-366.112287	-365.55746	-6.0
	TERT_AMINE_OTHER	na	na	-4.7 [d]<br>(-24.6)[b,e]
54	ETHER	-154.990462	-154.432513	-4.1
55	O_HYDROXYLAMINE	-249.575938	-249.016661	-3.2
56<br>57	TERT_AMINE_DIALKYL	-174.401695<br>(-174.848765)	-173.838692<br>(-174.323526)	-0.9<br>(-24.6)[b]
58	HYDROXYL	-115.717841	-115.154117	-0.4
	PROTON	-	-	0.0
59<br>60	PRIM_AMINE	-95.838644<br>(-96.283949)	-95.270678<br>(-95.754809)	2.2<br>(-22.1)[b]
61<br>62	SEC_AMINE_NONACIDIC	-135.120525<br>(-135.568378)	-134.551865<br>(-135.040243)	2.7<br>(-22.8)[b]
	SEC_AMINE_OTHER	na	na	2.7[f]<br>(-22.8)[b,f]
63	ALKYL	-79.788918	-79.219988	2.8
	REFERENCE COMPOUND (Methane)	-40.498890	-39.934456	0

a) for acidic group in its deprotonated form
b) for basic group in its protonated form
c) average of all 6-membered aromatic ring types
d) average of all tertiary amine types
e) equivalent to protonated form of dialkyl tertiary amine
f) equivalent to sec_amine_nonacidic
g) equivalent to sec_amide_nonacidic
h) average for all tertiary amide types

S9. Quantum mechanical energies for full QM method (structures provided as a xyz file, apart from for 1, 2 and 3 which are available elsewhere[3])

	Compound	$G_{neutral}$	G_{anion}	Change to ΔG_{deprot}
1	**1a**	-707.342232	-706.858418	-50.6
2	**1b**	-782.576559	-782.089858	-48.8
3	**1c**	-746.63647	-746.151287	-49.7
4	**1d**	-806.591982	-806.108372	-50.7
5	**1e**	-1166.94732	-1166.465696	-52.0
6	**1f**	-806.593491	-806.113592	-53.0
7	**1g**	-1166.948348	-1166.469328	-53.6
8	**1h**	-1044.403854	-1043.927444	-55.2
9	**2**	-985.258608	-984.775482	-51.0
10	**3a**	-1028.124167	-1027.649659	-56.4
11	**3b**	-1028.124896	-1027.643597	-52.2
12	**4a**	-646.994556	-646.506925	-48.2
13	**4b**	-746.245177	-745.758642	-48.9
14	**4c**	-761.496516	-761.008371	-47.9
15	**4d**	-647.179285	-646.691342	-48.0
16	**4e**	-491.217601	-490.735197	-51.5
17	**4f**	-663.043466	-662.558143	-49.6
18	**4g**	-664.220823	-663.73134	-47.0
19	**5a**	-686.283707	-685.793407	-46.5
20	**5b**	-785.53466	-785.045289	-47.1
21	**5c**	-800.785467	-800.293506	-45.5
22	**5f**	-702.3325	-701.843898	-47.6
23	**6**	-607.700245	-607.228721	-58.3
24	**7a**	-891.5712	-891.10031	-58.7
25	**7b**	-1122.56583	-1122.093508	-57.8
26	**7c**	-1254.121257	-1253.647519	-56.9
27	**7d**	-1368.345993	-1367.872777	-57.2
28	**8**	-1200.931405	-1200.462086	-59.7
29	**9**	-637.047081	-636.58012	-61.2
30	**10**	-479.906028	-479.428216	-54.4
31	**11**	-859.725165	-859.255269	-59.3
32	**12**	-1104.328218	-1103.855019	-57.3
33	**13**	-911.788586	-911.309436	-53.5
34	**14**	-416.002385	-415.513688	-47.5
35	**15**	-1425.180812	-1424.700557	-52.8
36	**16**	-1683.15954	-1682.695366	-62.9

S10. Explanation for why k_{obs} is maximized when pH ~ pKa

The rate law for general-base catalysis is given by Equation S8.1

$$k_{\text{obs}} = k_{\text{o}} + \sum k_{2,\text{gb}} \times f_{\text{basic}} \times [\text{buffer}] \qquad \text{Equation S7.1}$$

Here k_{obs} is the observed pseudo-first-order rate constant for the reaction, k_o is the first-order rate constant for the combined uncatalysed an hydroxide-catalysed reactions, and the summation is over the product of second-order rate constants ($k_{2,gb}$), the fraction of the buffer that is in the basic form (f_{basic}), and the buffer concentration [buffer].

The Brønsted relationship predicts the variation of $k_{2,gb}$ with pK_a while the Henderson-Hasselbalch equation predicts the variation of f_{basic} with pK_a for constant pH. Figure S5 illustrates the behaviour of f_{basic} and relative second-order rate constants ($k_{2,gb,rel}$; rate constants relative to a rate constant chosen to be 1 M^{-1} s^{-1} for a general-base catalyst with a conjugate acid of pK_a of 7 and assuming a Brønsted β of 0.5) for a solution pH of 7.

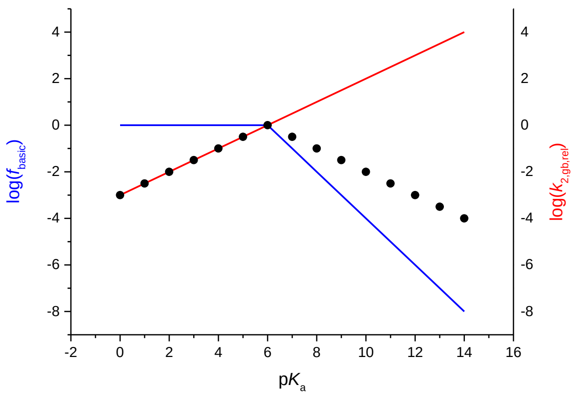

Figure S5: log(f_{basic}) (blue line), log($k_{2,gb,rel}$) (red line) and the resulting log($k_{2,gb,rel} \times f_{basic}$) as a function of p$K_a$ for a solution pH of 7.

Figure S5 shows that log($k_{2,gb,rel} \times f_{basic}$), and thus $k_{2,gb,rel} \times f_{basic}$, goes through a maximum at pK_a = pH.

S11. Frequency of occurrence of functional group types adjacent to chiral centres in the GOSTAR database.

Group	Number of occurrences adjacent to stereogenic centres in GOSTAR database
ALKYL	9504164
PROTON	4800487
REVERSE_SEC_AMIDE	1095576
SEC_AMIDE_NONACIDIC	837327
HYDROXYL	783878
ETHER	700116
BENZENE	607734
REVERSE_TERT_AMIDE	333774
ALKENE	315895
TERT_AMIDE_DIALKYL	214658
TERT_AMINE_DIALKYL	209581
REVERSE_ESTER	191227
FIVE_MEM_AROMATIC	167621
CARBOXYLIC_ACID	158356
KETONE	157598
SEC_AMINE_NONACIDIC	152486
PRIM_AMINE	121936
AROMATIC_ETHER	111082
ESTER	109326
OTHER	81503
SEC_AMIDE_ACIDIC	64619
SEC_AMINE_OTHER	57662
SEC_AMINE_ACIDIC	54030
TERT_AMINE_OTHER	53682
THIOETHER	52885
PRIM_AMIDE	47736
TERT_AMIDE_OTHER	41198
TERT_AMINE_ALKYL_AROMATIC	38354
IMINE	34178
EPOXIDE	31950
TERT_AMIDE_ALKYL_AROMATIC	28140
SIX_MEMBERED_GENERAL	26547
HALOGEN	23249
REVERSE_IMINE	22745
PYRIDINE_META	22501
SEC_AMIDE_OTHER	21411
HYDROXAMIC_ACID	19851
PYRIDINE_ORTH	17340
NITRILE	13081
ALKYNE	12460

O_HYDROXYLAMINE	11952
ETHER_DB	10200
PYRIDINE_PARA	9256
IMIDE	7588
QUAT_AMMONIUM	5668
PYRIMIDINE_2_6	4261
PYRIMIDINE_2_4	3652
PYRIMIDINE_3_5	2685
REV_AMIDE_DB	2146
PYRAZINE	1510
TERT_AMINE_DIAROMATIC	1472
THIOCARBONYL	1395
TERT_AMIDE_DIAROMATIC	1339
NITRO	616
PYRADIZINE_2_3	505
PYRADIZINE_3_4	306
TRIAZINE	100
ISONITRILE	37

S12. Justification of the cross-conjugation correction.

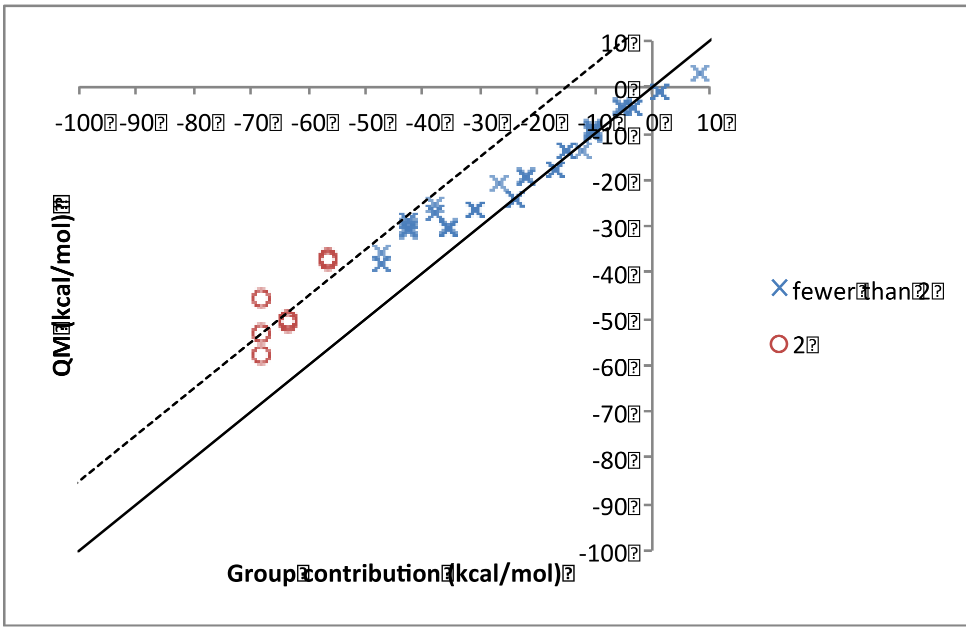

Figure S6. ΔΔG(R_1,R_2,R_3) plotted against ΣΔΔG for a set of compounds in which the number of groups that can stabilize an anion via conjugation (as indicated in section S7) is indicated.

The deprotonation energy computed via QM is plotted against the group contribution based deprotonation energy in Figure S6 for all combinations of alkyl, ether, secondary amide and reversed secondary amide (structures are generated combinatorially such that all values are at least duplicated). Compounds with two secondary amide groups are shown as red circles. The dashed line corresponds to the cross-conjugation correction of +15 kcal/mol.

S13. References

1 H. Fukada and K. Takahashi, *Proteins Struct. Funct. Bioinforma.*, 1998, **33**, 159–166.
2 A. A. Green, *J. Am. Chem. Soc.*, 1933, **55**, 2331–2336.
3 A. Ballard, Kinetics and mechanism of H/D exchange reactions and racemisation in aqueous solutions: configurational stability of ester and amide arylgycine derivatives, Cardiff University, 2011.
4 The pK_a of $H_2PO_4^-$ was assumed to be 7.2 in this study.
5 S. Narduolo, The mechanism of racemisation of 5-substituted hydantoins in aqueous solution, Cardiff University, 2011.
6 M. Zief and J. T. Edsall, *J. Am. Chem. Soc.*, 1937, **59**, 2245–2248.
7 M. Reist, P.-A. Carrupt, B. Testa, S. Lehmann and J. J. Hansen, *Helv. Chim. Acta*, 1996, **79**, 767–778.
8 S. P. Agarwal and M. I. Blake, *J. Pharm. Sci.*, 1968, **57**, 1434–5.
9 H. O. Ahmad, Kinetics and mechanism of racemisation reactions of configurationally labile stereogenic centres in drug-like molecules in aqueous solutions; thiohydantoins and related compounds., Cardiff University, 2015.
10 W. I. Congdon and J. T. Edward, *Can. J. Chem.*, 1972, **50**, 3780–3788.
11 Ahmad, H. O. Unpublished results.
12 M. Reist, L. H. Christiansen, P. Christoffersen, P.-A. Carrupt and B. Testa, *Chirality*, 1995, **7**, 469–473.
13 B. Mey, H. Paulus, E. Lamparter and G. Blaschke, *Chirality*, 1998, **10**, 307–315.
14 M. Brandl, D. Conley, D. Johnson and D. Johnson, *J. Pharm. Sci.*, 1995, **84**, 1045–1048.
15 B. Jamali, I. Bjornsdottir, O. Nordfang and S. H. Hansen, *J. Pharm. Biomed. Anal.*, 2008, **46**, 82–87.
16 A. A. A. Boraei, *Phosphorus Sulfur Silicon Relat. Elem.*, 1998, **142**, 69–81.
17 M. Reist, P. A. Carrupt, E. Francotte and B. Testa, *Chem. Res. Toxicol.*, 1998, **11**, 1521–1528.
18 E. Freese, B. C. Levin, R. Pearce, T. Sreevalsan, J. J. Kaufman, W. S. Koski and N. M. Semo, *Teratology*, 1979, **20**, 413–440.
19 C. Danel, C. Foulon, J.-F. Goossens, J.-P. Bonte and C. Vaccher, *Tetrahedron Asymmetry*, 2006, **17**, 2317–2321.
20 A. Avdeef, *J. Pharm. Sci.*, 1993, **82**, 183–190.
21 M. Reist, M. Roy-de Vos, J. P. Montseny, J. M. Mayer, P. A. Carrupt, Y. Berger and B. Testa, *Drug Metab. Dispos. Biol. Fate Chem.*, 2000, **28**, 1405–1410.
22 http://products.sanofi.ca/en/plavix.pdf [accessed 30th January 2017].
23 A. K. Nair, S. Balla, V. Jayaramreddy, C. S. Kandi and P. S. Reddy, *Int. J. Sci. Innov. Discov.*, 2012, **2**, 351–358.
24 E. Bosch, P. Bou, H. Allemann and M. Rosés, *Anal. Chem.*, 1996, **68**, 3651–3657.
